# Factors Associated with Eating in the Absence of Hunger among Children and Adolescents: A Systematic Review

**DOI:** 10.3390/nu14224715

**Published:** 2022-11-09

**Authors:** Catherine Savard, Stéphanie Bégin, Véronique Gingras

**Affiliations:** 1 Department of Nutrition, Université de Montréal, Montreal, QC H3T 1A8, Canada; 2 Research Center of the Centre Hospitalier Universitaire Sainte-Justine, Montreal, QC H3T 1C5, Canada

**Keywords:** eating in the absence of hunger, dietary behavior, children, adolescents, factors, obesity, nutrition, correlates

## Abstract

Eating in the absence of hunger (EAH) has been extensively studied over the past two decades and has been associated with excess body weight and the development of obesity. However, determinants of EAH remain uncertain. This systematic review aims to identify individual, familial, and environmental factors associated with EAH among children and adolescents. We included studies with a measure of EAH in participants aged 3–17 years old and including ≥1 factor associated with EAH. Our search identified 1494 articles. Of these, we included 81 studies: 53 cross-sectional, 19 longitudinal and nine intervention studies. In childhood (≤12 years old), EAH increases with age, it is greater in boys compared to girls, and it is positively associated with adiposity. Moreover, EAH development seems to be influenced by genetics. In adolescence, the number of studies is limited; yet, studies show that EAH slightly increases or remains stable with age, is not clearly different between sexes, and findings for overweight or obesity are less consistent across studies in adolescence. For familial factors, parental restrictive feeding practices are positively associated with EAH during childhood, mostly for girls. Studies assessing environmental factors are lacking and robust longitudinal studies spanning from early childhood to adolescence are needed.

## 1. Introduction

Eating in the absence of hunger (EAH), i.e., eating past the point of satiety, is a behavior described for the first time by Fisher and Birch in 1999 [1]. They assessed this behavior in a laboratory setting to obtain an operationalized measure of EAH, which is now considered the reference method to assess EAH [2,3]. During the assessment, the subject is first instructed to eat a meal until satiety (pre-load phase). Then, the subject is left alone in a room with various age-adapted toys or games and pre-weighted portions of palatable snacks that are available to eat in free access for 10 min (free-access phase). After this second phase, the leftover snacks are weighed to assess the consumed amount, which is converted into kilocalories (kcals). This absolute value of kcals represents the level of EAH.

The protocol to assess EAH is time consuming and requires important resources. To facilitate the assessment of this behavior, an EAH questionnaire for children and adolescents (EAH-C) has been developed by Tanofsky–Kraff and collaborators [4]. This 14-item questionnaire assesses EAH globally, but also in response to three specific triggers; negative affect, external cues and fatigue/boredom [4]. Good convergent validity [4,5], as well as internal consistency and temporal stability [4], have been demonstrated for the EAH-C questionnaire. A parent-report version of the questionnaire (EAH-P) is also available [6].

EAH has been associated with excess body weight and with the development of obesity [2]. Childhood obesity is a major public health concern; the worldwide prevalence of childhood obesity is four times higher than in 1975, with 18% of children aged 5–19 years old that currently have overweight or obesity [7]. In this context, identifying modifiable risk factors for childhood obesity, such as eating behaviors, is of crucial importance [8]. We thus believe that interventions to curb the development of EAH could be beneficial to reduce childhood obesity; yet, we do not know who would benefit from such interventions since many uncertainties persist regarding the determinants of EAH. For example, we know that EAH can be observed in children as early as three years old and it is known to become more important with advancing age [2]. Infancy and early childhood could thus be a window of opportunity to prevent the development of EAH, but perinatal factors associated with this behavior are poorly understood, thus hindering our ability to determine potential prevention targets.

EAH has been extensively studied over the past two decades and to our knowledge, only one systematic review of EAH determinants has been conducted, and in children only [2]. Lansigan and collaborators restricted their review to studies examining EAH with the laboratory protocol and to participants ≤ 12 years old, and included 19 studies published until 2013 [2]. The main conclusions from their review were: (1) EAH can be observed in boys and girls; (2) EAH increases with age; (3) EAH is positively associated with weight status; (4) EAH has a genetic component; (5) maternal feeding practices are associated with EAH among girls. However, no systematic review assessing EAH in adolescence has been published. In addition, the EAH-C questionnaire has been increasingly used over the past years to study EAH in large cohorts. Consequently, this systematic review aims to explore factors associated with EAH among children and adolescents by including both assessment methods of EAH: the laboratory protocol and the EAH-C/EAH-P questionnaires. We will explore sex differences in the correlates of EAH, as well as regarding the differences in individual and familial factors in adolescence compared to childhood.

## 2. Materials and Methods

This systematic review has been registered on PROSPERO (registration number CRD42021254624) and we conducted our review following the Preferred Reporting Items for Systematic reviews and Meta-Analyses (PRISMA) guidelines.

### 2.1. Search Strategy

A research librarian performed a systematic literature search of the following databases: Web of Science, Cochrane Library, CINAHL, PsycINFO, Google Scholar, CAB abstracts (search terms available in Supplementary Materials). The last search was conducted on 14 June 2021. We did not restrict our search based on studies’ dates of publication.

### 2.2. Eligibility Criteria

We included randomized and non-randomized intervention studies, as well as observational studies. We only included peer-reviewed publications in English or French unless a translated version was available. Studies were included if they assessed EAH with the laboratory protocol or with the EAH-C or EAH-P questionnaires. For the EAH paradigm, we did not restrict the eligible studies based on how EAH was operationalized (e.g., total kcals, weight (g), percent of daily energy requirements). Additionally, we included studies in which the pre-load was a meal (e.g., breakfast, lunch, dinner); studies with snacks or a drink as pre-load were excluded. The eligible studies evaluating self-report or parent report of their child’s EAH needed to use the original EAH-C or EAH-P questionnaires [4,6] or the questionnaires’ specific subscale(s) or question(s).

We limited the eligible studies to human participants, specifically children aged 3 to 17 years old. We also included studies with overlapping age range if the mean age of the sample was between 3 and 17 years old or if they had a separate analysis with subgroups within this age range. Studies among children with a medical condition potentially affecting satiety and hunger or with a diagnosed eating disorder were excluded. Furthermore, the eligible studies needed to measure one of the three selected domains of factors associated with EAH: individual (e.g., sex, age, race/ethnicity, physical activity level), familial (e.g., parental feeding practices, parents’ demographics) or environmental (e.g., school setting and policies, exposure to food publicity).

### 2.3. Screening

The results of our searches were imported into the Covidence software (Veritas Health Innovation, Australia. Available at www.covidence.org (accessed on 10 June 2021)) where duplicates were removed. Then, CS and SB independently reviewed, based on the eligibility criteria, all titles and abstracts of the articles and screened the full text of all the potentially eligible articles. Any conflicts for abstract or full-text screening were resolved by a third reviewer (VG). References from the included studies were also manually screened.

### 2.4. Quality Assessment

Quality assessment was performed by CS using a quality rating scheme previously adapted from the Cochrane guidelines on quality assessment by Mikkelsen et al. [9]. Accordingly, studies could be rated as weak, moderate, strong or very strong based on the extent of details provided in the study, sample size, study duration, methodological flaws and study design. SB also independently reviewed the quality assessment.

### 2.5. Data Extraction and Synthesis

Data extraction was performed by one reviewer (CS) and verified for accuracy by a second reviewer (SB). Any disagreement was resolved through discussion with VG. We created a predetermined template for data extraction including the following information: authors and date of publication, source(s) of research funding and potential conflicts of interest, study design and duration, study context and sample size, EAH assessment method, as well as individual, familial and environmental characteristics associated with EAH. Detailed characteristics for each included study are available in Tables S1–S5 in Supplementary Materials.

We could not perform a meta-analysis, notably because of the large variability in assessment methods (EAH and correlates’ assessments). Studies are thus presented as a narrative synthesis, organized in three sections for the categories of factors (individual, familial or environmental). Within each section, we synthesized the results by factor, by children (≤12 years) vs. adolescents (>12 years), and we looked at sex/gender differences in findings. We decided to present results for children and adolescents separately since adolescence is a particular period of development and correlates of eating behaviors during this period might differ from childhood.

## 3. Results

**Figure 1:**
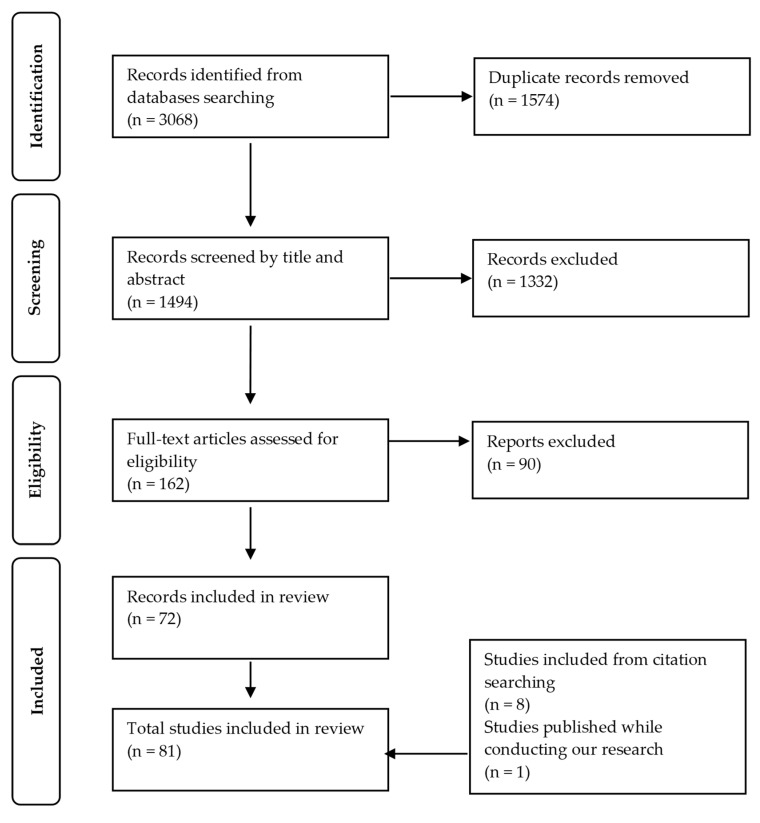
A PRISMA flow diagram of the screening process.

We retrieved a total of 1494 articles (duplicates removed) from the databases. The PRISMA flow diagram of the screening process is presented in Figure 1. At the end of the process, 81 studies were included in our narrative synthesis. Of these 81 studies, 53 were cross-sectional, 19 were longitudinal and nine were intervention studies. Some longitudinal studies included cross-sectional analyses. The ages of participants in these studies varied from 33 months to 25 years of age (mean age for all studies was within the range of 3 to 17 years old as defined in our inclusion criteria); 61 studies were among children and 20 studies were among adolescents (one study included subgroup analyses for both periods). Most of the included studies assessed EAH with the laboratory protocol (n = 65) compared to the questionnaire (n = 14), with two studies using both assessment methods. Studies were primarily conducted in the United States (n = 57), while the other studies were conducted in Europe (n = 9), Chile (n = 7), Singapore (n = 4), Australia and New-Zealand (n = 4). Detailed characteristics of individual studies can be found in Tables S1–S5 in Supplementary Materials. A summary of overall conclusions for individual and familial factors, by age period (childhood vs. adolescence) are presented in Table 1 (individual factors) and Table 2 (familial factors).

### 3.1. Study Quality Assessment

A quality assessment of included studies is presented in Tables S1–S5 in Supplementary Materials. Most of the studies (68%) were rated either strong or very strong. Studies rated as of moderate quality (30%) had a small sample size, missing details regarding participants’ characteristic (e.g., ethnicity, sex/gender, etc.) and/or methodological flaws in the EAH protocol (e.g., inclusion of children who were not full after the pre-load meal, had longer delay between the pre-load meal and the free access phase, had a limited number of palatable snacks available, etc.).

### 3.2. Individual Factors Associated with EAH

#### 3.2.1. Age

As previously reported by Lansigan et al., EAH has been observed in children as early as 3 years old [2], and even slightly younger in a United States (U.S.) cohort beginning at 33 months of age [10]. Twenty-two studies examined the association between age and EAH among children. In cross-sectional analyses, six [11,12,13,14,15,16] out of 13 studies reported a positive association between age and EAH. In a cohort of children aged 5–18 years old, EAH increased with age until 13 years old and then stabilized [11]. On the other hand, five studies did not find any association between age and EAH [17,18,19,20,21]. In prospective cohort studies, seven [22,23,24,25,26,27,28] out of 9 studies reported that EAH increased and two reported that it was stable as children got older; however, six of these studies are from the same cohort of girls who were followed from 5 to 13 years old [3,22,23,24,25,27]. Age was also identified has a moderator for other correlates of EAH in several studies, as discussed in respective sections [15,16,22,23,24,25,27]. 

EAH has also been observed in adolescents [24,29,30,31,32,33,34,35,36,37,38,39,40,41,42,43,44,45,46,47,48]. In cross-sectional studies, one study showed that EAH was positively associated with age [42], although another study found no association [40]. Only one longitudinal study reported that EAH was similar throughout adolescence [32]. Age was also a moderator for other associations in one study [45].

#### 3.2.2. Sex/Gender

None of the studies included in this review addressed gender specifically, and thus we report findings associated with sex as a biological/physiological correlate. Most studies are from mixed-sex cohorts, 12 studies are from cohorts with girls only and 2 did not report the sex distribution of their population [36,49]. Overall, 20 studies examined the associations of sex and EAH among children. Of these, ten reported that boys engaged in more EAH than girls [11,12,14,17,19,50,51,52,53,54]. One study, conducted in a sample of children with excess weight or obesity, showed that EAH was greater in girls compared to boys [18]. Amongst the nine studies which found no association, four [1,55,56,57] were conducted in small samples (n ≤ 75) of younger children (3 to 6 years old) and two assessed EAH using the EAH-C [58] or the EAH-P [20] questionnaires. 

In adolescents, seven studies reported associations of child’s sex and EAH, and findings are discordant. In two studies using data from the same cohort, Kelly et al. showed greater EAH in boys compared to girls [32,33]; yet in their study with a prospective (one-year) follow-up, this association was only observed at the baseline assessment [32]. On the other hand, two studies showed greater EAH in girls compared to boys [42,43], while another study showed that girls had higher EAH scores than boys for the negative affect subscale of the EAH-C questionnaire only [48]. Two more studies did not find associations of sex and EAH in adolescents [40,46].

Many studies also addressed sex as a moderator or as a mediator for associations of EAH and other factors, including child’s weight status [16,21,32,35,53,56,59,60] or parental feeding practices [1,51,55]; these effects are presented in the factors’ respective sections.

#### 3.2.3. Adiposity

The association between adiposity and EAH in children has been extensively studied (n = 31 studies). Most studies assessed adiposity as body mass index (BMI) percentile and z-score adjusted for age and sex, with overweight status defined as BMI ≤ 85th percentile. In cross-sectional analyses, 15 [11,12,14,16,17,19,20,28,53,54,56,58,60,61,62] out of 23 reported a positive association between adiposity and EAH, including two studies where the association was only observed in girls [56,60] and one study where the association was only observed in boys [53]. Another study where weight status was positively associated with EAH found weight status-by-sex and by-age interactions; older siblings with excess weight/obesity engaged in more EAH than older and younger siblings with normal-weight, and boys and girls with excess weight/obesity engaged in more EAH when compared to girls with normal-weight [16]. A different study found a positive association between adiposity and EAH (fatigue/boredom and external cues subscales) only in individuals with a 16p11.2 chromosomal deletion [20]. Only two studies reported a negative association between adiposity and EAH, and among girls only [21,53]. The other studies did not find associations between adiposity and EAH [18,50,57,63,64,65,66]. In prospective analyses, all five studies [3,22,23,25,26] reported a positive association between adiposity and EAH, although four of these studies were conducted in the same cohort of girls in the U.S. who were followed from 5 to 9 years old [3,22,23,25]. These studies mainly showed that adiposity at 5 years old was associated with a greater increase of EAH over the years [25], and one study found a greater increase of EAH among girls of mothers with excess weight [23]. Adiposity at 5 years old was also positively associated with EAH at 7 years old [3,25] and 9 years old [25]. The other study showed that adiposity was associated with higher EAH 18 months later [26]. The association between children’s birthweight z-score and EAH was examined in two prospective cohort studies [10,57]; one study found no association [57] and the other study found that a higher or a lower birthweight z-score were associated with more EAH, for girls only [10]. Adiposity was also a moderator of associations between EAH and other correlates [54,59].

Fewer studies reported on associations between adiposity and EAH in adolescents (n = 9), and some studies assessed body composition in addition to BMI. Among five cross-sectional studies, two studies showed that adiposity was positively associated with EAH [40,45], an association that was stronger for younger participants (<13 years old) in one study [45]. Two studies reported a negative association between weight status and EAH [31,35], but only for girls in one of these studies [35]. Associations of body composition and EAH were also examined, and percentage of body fat has been positively associated with EAH [40], while fat mass [41] and fat-free mass [40] were not associated with EAH. Another study found a positive association between fat-free mass and EAH in a cross-sectional analysis [33]; however, longitudinal data from this same cohort showed that although baseline adiposity and adiposity one year later were associated with EAH, no associations were found for changes in adiposity over the one year period and EAH [32]. Similarly, Derks et al. showed that in cross-sectional analyses, adiposity, fat mass and fat-free mass were positively associated with EAH; however, when looking prospectively at associations of body composition and EAH, no associations were found [47]. Finally, overweight/obesity status was not associated with EAH in a cross-sectional analysis of a Chilean cohort [46].

#### 3.2.4. Exposure to Breastfeeding

In children, two prospective analyses found no association for exposure to breastfeeding and the development of EAH [57,67]. In one study examining the association of exposure to breastfeeding and EAH in adolescents (mean age: 16.7 years old), EAH was less pronounced in participants who had been exclusively breastfed for >6 months as infants [46]. 

#### 3.2.5. In utero Exposure to Maternal Glucose Intolerance

Two prospective studies examined associations of in utero exposure to maternal diabetes and EAH assessed with the EAH-C questionnaire in adolescents [47,48]. Shapiro et al. showed that exposure to gestational diabetes (GDM) was associated with a higher EAH-C total score and a higher score on the fatigue/boredom subscale in girls, while no associations were found in boys [48]. Derks et al. found no association between exposure to GDM and EAH, but in their cohort, in utero exposure to maternal glucose intolerance (below the diagnostic threshold for GDM) was positively associated with EAH in girls and negatively associated with EAH in boys [47].

#### 3.2.6. Genetics

A total of nine studies investigated the influence of genetics on EAH in children [11,16,17,20,36,59,68,69,70] and one in young adolescents [58]. Fisher et al. demonstrated heritability of EAH among Hispanic families [11] and Kral et al. observed that EAH was similar between full siblings, but not between half-siblings [16]. The association between the rs9939609 polymorphism of the FTO gene and EAH was examined in three studies [17,59,68]. Wardle et al. showed that children carrying the TT genotype engaged in less EAH than children carrying the A allele [68]. Similarly, the FTO genotype modified the effect of food advertisement exposure on EAH, with the AT and AA genotypes being associated with greater EAH [17]. These findings are also consistent with a third study where carriers of the A allele reported more frequent EAH due to negative emotion compared to non-carriers, although this association was observed in boys with normal weight only [59]. In a similar Chilean cohort, two studies investigated the association between the rs1800497 polymorphism of the dopamine D2 receptor gene and EAH, and no associations were reported for children’s [36] or young adolescents’ genotype and EAH [58]. The relationship between the rs17782313 polymorphism of the melanocortin-4 receptor gene and EAH was reported in two studies. In the first study, self-reported EAH was not associated with children’s genotype [70], while in the second study, carriers of the C allele engaged in more EAH than non-carriers, although the association did not reach significance (only ten participants had a measure of EAH) [69]. Finally, one study from a U.S. cohort showed that carriers of a 16p11.2 deletion engaged in more EAH due to external cues and due to boredom only (EAH-C questionnaire) [20]. 

#### 3.2.7. Eating Behaviors

Among the 13 studies assessing children’s eating behavior in children, four used the Children Eating Behavior Questionnaire (CEBQ) [10,12,14,57]. Food responsiveness and satiety responsiveness [10,12,14,57], enjoyment of food [10,57], slowness in eating [57] and emotional overeating [14] were assessed, and no associations were found for these eating behaviors and EAH in most studies [12,14,57]. One study used the Dutch Eating Behavior Questionnaire (DEBQ) and found that external and emotional eating, but not restrained eating, were positively associated with EAH [21]. Appetitive self-regulation (delay of gratification task) was assessed in four studies [12,13,60,71], of which three showed positive associations between EAH and appetitive self-regulation [12,13,71], including one prospective analysis between appetitive self-regulation and EAH one year later [71]. In this study, children with both lower inhibitory control and lower appetitive self-regulation had the highest EAH level one year later [71]. In the fourth study, EAH was positively associated with the relative reinforcement value for food, but not with the ability to delay gratification, and in girls only [60]. Fisher and Birch reported no association of girls’ negative self-evaluation of eating (reporting eating too much and reporting having negative feelings about eating) and EAH [72]. Two studies found no associations between EAH and caloric compensation index [16,19]. Fisher et al. showed that children’s response to meal size (larger food intake when served a larger entrée) was associated with greater EAH, but no association was found for bite frequency and size, as well as self-served portion size [73]. Finally, Fogel et al. showed no association of EAH with children’s selection of larger ideal portion sizes [65]. 

In adolescents, associations of eating behaviors and EAH were assessed in six cross-sectional studies [30,33,37,39,42,45]. One study showed that dietary restraint was positively associated with EAH in girls only [33]. In another study, EAH was positively associated with emotional eating [42]. For binge-eating and loss of control (LOC) eating, one study found no association [30] and three studies reported positive associations between binge-eating and/or LOC eating and EAH [37,39,45]. In the study from Zocca et al., the positive association observed between LOC eating and EAH was stronger for pre-adolescents (<13 years old) compared to adolescents (≥13 years old) [45]. In the study from Radin et al., only state LOC eating (during the pre-load meal of the EAH test), and not past month’s self-reported LOC eating, was associated with EAH [37]. Finally, Shomaker et al. showed that objective and subjective binge eating (LOC eating with or without objectively large amount of consumed food) were associated with greater EAH when compared to overeating without LOC eating or to the absence of overeating episodes [39]. 

#### 3.2.8. Neurobehavioral Measures

Nine studies reported neurobehavioral measures in children [12,15,20,27,28,52,61,71,74], with most cross-sectional analyses showing that dimensions of children’s temperament were not associated with EAH. This includes assessments of inhibitory control [12,27,52], effortful control [12,74], cognitive flexibility [12], positive affect [12], and impulsivity [15]. However, Fogel et al. reported that children who had a restless behavior had a higher EAH intake compared to those who had typical behavior during the assessment of inhibitory control [52]. Intelligence quotient was not associated with EAH in a cohort of individuals with a specific 16p11.2 genotype [20]. Moreover, negative affect was inversely associated with EAH, and surgency (a dimension of approach) was positively associated with EAH [74]. In another study, approach was not associated with EAH, in a cohort of girls [27]. After children undertook a stress induction protocol, EAH was positively associated with observed anxiety in one study [61] and with higher cortisol release in response to the stressor, but only for older children (8–9 years old) in another study [15]. In a prospective cohort study, overall child exposure to psychological stress predicted a higher increase in EAH over time [28]. However, when examined individually, stress exposure from proximal parenting, family-level functioning and contextual factor were not associated with EAH [28]. In another prospective analysis, neither inhibitory control nor attentional control [71] were associated with EAH. However, children with both lower inhibitory control and low appetitive self-regulation (delay of gratification task) had the highest EAH intake 1 year later [71]. 

In two studies in adolescents, emotional regulation was associated with EAH [42,43]. More precisely, negative affect [42] and alexithymia (inability to recognize emotions) [43] were associated with a higher level of self-reported EAH. Depressive symptoms were not associated with EAH in another study [33]. Dispositional mindfulness was assessed in two studies of adolescent girls, one of which showed an inverse association of mindfulness and measured EAH [30], while the second study also found an inverse association for mindfulness and self-reported EAH due to fatigue/boredom [44].

#### 3.2.9. Lifestyle Habits

Six studies (two in children and four in adolescents) investigated the associations between lifestyle and EAH [17,34,41,47,48,75]. Screen time and physical activity were assessed in one study and both habits were not associated with EAH [17]. Association of sleep patterns and EAH in children was also examined and no associations between intervention-induced sleep extension or reduction and subsequent EAH were found [75]. 

In adolescents, mixed findings were reported for associations of sleep and EAH. In a sample of teenage girls at risk for type 2 diabetes, daytime sleepiness and sleep duration were not associated with EAH [34]. In another study, no association was found for weekly sleep duration and bedtime; yet, longer sleep duration on weeknights was associated with less EAH, while longer sleep duration and longer catch-up sleep duration on weekends were associated with more EAH [41]. Two studies assessed eating habits in adolescents and reported a positive association between daily energy intake [48] and sugar-sweetened beverages intake [47] with EAH, but no association with low nutritive value food intake [47].

#### 3.2.10. Perceived Sociocultural Pressures and Body Image

The relationship between perceived sociocultural pressures or body image and EAH has been assessed in two studies conducted among adolescents [38,42]. In a first study, Reina et al. showed that perceived pressure to be thin from family was positively associated with EAH, while pressure to be thin from the media and the use of media for information about beauty ideals were positively associated with EAH in girls only, and no associations were found for pressure from friends [38]. Appearance orientation and preoccupation with excess weight gain (two dimensions of body image) mediated the associations of perceived sociocultural pressures with EAH [38]. In a second study, by Rubin et al., child-reported weight-based teasing was associated with a higher level of EAH and this association was mediated by negative affect [42].

#### 3.2.11. Emotional State (Affect)

Two studies used a negative mood induction protocol (doing a puzzle with a missing piece [76] and missing a crayon to complete a drawing [66] to induce mild stress) while assessing EAH in children. In a cohort of young children (5–7 years old), exposure to a mild stressor (negative mood induction) resulted in greater EAH [66]. However, in a cohort of slightly younger children (3–5 years old), there was no significant effect of a negative mood induction on EAH [76]. 

In adolescents, two studies (same cohort) showed no effect of inducing a sad mood, compared to a neutral mood, on EAH [33,37].

#### 3.2.12. Brain Activity Measures

The associations of brain activity and EAH have been assessed in four studies conducted among children [50,63,64,77]. Neuronal activity in regions of the reward network of the brain was associated with greater EAH in three studies [63,64,77]. In one of these studies, conducted in a small sample of 10 children with obesity and 13 children with normal weight, a positive trend was observed only among children with obesity [64]. In another study, an increased brain response to food compared to money rewards, in brain regions responsible for inhibitory control or rewards, was positively associated with EAH [50]. 

#### 3.2.13. Other Individual Factors

Eight studies investigated other individual factors. In a study among children, EAH was not associated with race/ethnicity [54]. While one study reported that EAH was positively associated with pubertal status [61], another one did not find any association between these two variables [50]. Fisher et al. found that EAH was positively associated with fasting leptin and insulin levels, but not with ghrelin and amylin levels [11]. In this same study, child acculturation was not associated with EAH [11]. In another study, being exposed to food publicity before the EAH free access phase was positively associated with EAH, but for the advertised snack only [17]. EAH was not associated with other perinatal factors including nutritive sucking [78] and child’s age at the time of solid food introduction [57]. In adolescents, EAH was not associated with race/ethnicity [40], but it was positively associated with pubertal status [40].

### 3.3. Familial Factors Associated with EAH

#### 3.3.1. Parental Adiposity

Ten studies investigated the association between parental adiposity and EAH in children. Three studies found no association between maternal [21,66] or parental adiposity [79] and children’s EAH. A positive association between EAH and maternal pre-pregnancy weight was found for boys only [78], while no association was found for gestational weight gain (adequate vs. excessive) and EAH in another study [10]. The only study that investigated an interaction by parental sex found that paternal BMI was not associated with EAH, while maternal BMI was positively associated with EAH via maternal disinhibition in girls only [56]. In another study, children with excess weight/obesity who had mothers with excess weight/obesity had greater EAH compared to children with a normal weight who had mothers with either a normal weight or with excess weight/obesity [54]. In prospective studies, three studies [23,24,27] found associations of parental BMI with children’s EAH; all three studies were from the same cohort of girls from the U.S. [23,24,27]. In these studies, cross-sectional analyses showed that lower maternal BMI was associated with more EAH at 5 years old [23,27]. However, between 5–13 years old, EAH increased more importantly in girls who had mothers with excess weight [23] or for whom both parents had excess weight [24]. Maternal BMI was also a moderator of associations between EAH and parental restrictive feeding practices and daughter’s adiposity, which is discussed in their respective paragraphs [23]. 

In adolescents, one cross-sectional study showed no association of maternal BMI with EAH at ~14 years old [45]. Another study examined associations of maternal pre-pregnancy BMI (authors categorized children of their longitudinal cohort as high-risk for obesity if mothers had a pre-pregnancy BMI >66th percentile and low-risk for obesity if maternal pre-pregnancy BMI was <33rd percentile) and EAH in adolescence, and this study showed that EAH was greater among low-risk girls compared to low-risk boys, high-risk boys and high-risk girls [35].

#### 3.3.2. Parental Eating Style

Self-reported parental eating style assessed using the Eating Inventory Questionnaire and its association with EAH in children was examined in two studies [24,56]. In a first cross-sectional study, maternal disinhibited eating style was positively associated with EAH in girls, but not in boys, and no association was found for maternal dietary restraint and children’s EAH [56]. Maternal disinhibition was also found to be on the pathway of the association of maternal BMI and girls’ EAH in this same study [56]. In a prospective cohort of girls, maternal disinhibited eating style when their daughters were 5 years old was associated with EAH between 9–13 years old [24]. In the two previously cited studies, paternal eating style was not associated with children’s EAH [24,56].

In adolescents, a cross-sectional study found a positive association between maternal binge eating and children’s EAH and this association was stronger for pre-adolescents (<13 years old) compared to adolescents (≥13 years old) [45]. Maternal EAH was also positively associated with children’s EAH and this association was stronger for adolescents [45].

#### 3.3.3. Parental Restrictive Feeding Practices

Overall, 19 of the included studies among children reported measures of parental restrictive feeding practices. Most studies assessed parental restrictive feeding practices with questionnaires such as the Child Feeding Questionnaire [3,17,18,19,20,21,22,23,27,38,51,55,66,76,80,81], while one study assessed parental feeding practices through observation [14] and two from the child’s perception [1,72]. In cross-sectional analyses, six out of 14 studies reported a positive association between parental use of restrictive feeding practices and children’s EAH [1,17,20,51,72,81]. Of these 6 studies, two were conducted among girls only [72,81], two found an association in girls but not in boys [1,51], and one study found an association only in individuals with a 16p11.2 chromosomal deletion and only for the fatigue/boredom and external cues subscales of the EAH-P questionnaire [20]. Findings from a prospective analysis were more consistent with all five studies reporting a positive association between parental restrictive feeding practices and EAH, although four of these studies [3,22,23,27] were conducted within the same cohort assessing maternal use of restrictive feeding practices at 5 years old. Mainly, these studies showed that maternal use of restriction was positively associated with EAH from age 5 to 9, but in girls from mothers with excess weight only [23], and that the highest increase in EAH over the years occurred in girls from a mother with a higher use of restrictive feeding practices and with excess weight [22] or with lower inhibitory control [27]. The other study where a positive association was reported showed that parental use of restrictive feeding practices when the child was 3–5 years old was positively associated with EAH two years later under conditions of negative emotion (in a mood-induction experiment) [66]. 

In a cross-sectional study among adolescents, a positive association was found between parental use of restrictive feeding practices and adolescents’ EAH, and this association was mediated by adolescents’ appearance orientation and preoccupation with excess weight gain [38]. 

#### 3.3.4. Other Parental Feeding Practices

The associations of other parental feeding practices and EAH were assessed in 11 studies among children. In five cross-sectional studies [18,19,21,76,80] and one prospective study [66], parental use of pressure to eat was not associated with EAH while two cross-sectional studies found a positive [55] and a negative [51] association between maternal use of pressure to eat and EAH among boys only. In another study, pressure to eat was negatively associated with EAH due to external cues and boredom only (EAH-P questionnaire) in individuals with a 16p11.2 chromosomal deletion [20]. In a prospective study, pressure to eat was negatively associated with EAH at baseline and 18 months later [26]. However, in another study, self-reported prompting to eat by parents was not associated with EAH [80]. One study reported that a higher parental monitoring of food was negatively associated with children’s EAH [80], while four studies investigating the same factor found no significant association with EAH [18,19,21,55]. Using food as a reward was not associated with EAH under mood induction conditions when children were 3–5 years old [76]; yet, in a subgroup of participants with longitudinal follow-up, use of food as a reward was positively associated with EAH two years later when children were subjected to a mild emotional stressor and negatively associated with EAH two years later under neutral conditions of the experiment [66]. Moderately allowing the child to serve himself during a meal was associated with greater EAH in one study [14] and other feeding practices including child control [19], covert control [51], using food to regulate emotion [19,66,76] and allowing choice [14] were not associated with EAH. A negative emotional state was also a moderator of associations of parental feeding practices and EAH in a cross-sectional study among young children [76].

In adolescents, other child-feeding practices (monitoring and concern) were assessed in one cross-sectional study and were positively associated with EAH, but only through body image mediators (overweight preoccupation) [38]. 

#### 3.3.5. Parental Demographics 

Some studies were conducted exclusively in participants from low-income families [12,13,28,61,74]. In other studies, the family’s socioeconomic position (Hollingshead Index of Social Position) [21], household food security (U.S. Department of Agriculture six-item Household Food Security Survey) [11,54] and family income [17,54] were not associated with EAH in children. Only one study showed that EAH due to external cues was inversely associated with household food security [82]. Moreover, EAH was not associated with maternal education [17,57,66] and maternal age at delivery [57]. 

#### 3.3.6. Other Familial Factors

Four studies addressed other familial factors during childhood. Parental behavior as perceived by children was assessed in one study and EAH was positively associated with maternal psychological control, but it was not associated with firm control and acceptance [18]. One study calculated a composite risk score for overweight in children based on a combination of parental (maternal pre-pregnancy excess weight and paternal excess weight, excessive gestational weight gain) and individual risk factors (exposure to elevated maternal fasting glucose during pregnancy, short breastfeeding duration and early introduction of solid foods) and found no association between this score and EAH among children [83]. Kral et al. found that EAH was lower among children from households with a higher obesogenic food availability, independently of household food security [54]. Moreover, EAH was not associated with home environment quality (level of chaos in home assessed by questionnaire) [74].

## 4. Discussion

This systematic review provides the first report of EAH correlates among both children and adolescents and including the two EAH assessment methods, i.e., the laboratory protocol [1] and the EAH-C/EAH-P questionnaires [4]. Together, the 81 studies included in this review examined over 20 different potential individual and familial factors of EAH. Although several discrepancies emerged for factors associated with EAH, studies quite consistently indicated that EAH increases with age, but tends to stabilize during adolescence. In addition, during childhood, EAH appears to be greater among boys and to be positively associated with adiposity. EAH also seems to have a genetic component. For familial factors, studies indicate that, during childhood, parental restrictive feeding practices are positively associated with EAH, mostly among girls.

We explored differences in correlates of EAH in adolescence compared to childhood. 

Although clear conclusions were difficult to draw, some discrepancies were found between correlates in childhood and adolescence. Overall, studies included in our review indicate that while EAH seems to increase with age during childhood, it tends to remain stable during adolescence. As opposed to findings in childhood, no consistent associations with EAH were found in adolescence for adiposity and sex. Also, associations of familial characteristics such as maternal eating style, parental adiposity and parental restrictive feeding practices with EAH were only observed in childhood or were more consistent during childhood. Yet, due to the absence or limited number of studies, as well as the low number of studies assessing factors associated with EAH longitudinally from childhood to adolescence, some differences between childhood and adolescence could not be assessed. In addition, while the physical environment in the community has been shown to influence adolescents’ eating behaviors [84], no study has yet assessed environmental factors associated with EAH in adolescents.

We also examined sex differences in EAH correlates. While EAH was observed in boys and girls, included studies suggest that EAH might be more pronounced in boys during childhood. No clear differences in EAH between boys and girls during adolescence were observed. Some factors associated with EAH were sex specific. Interestingly, the main differences we found were among familial factors. Our results showed that parental use of restrictive feeding practices was associated with EAH, mostly in girls. However, these results need to be interpreted carefully since evidence was partially issued from the same cohort of girls followed from age 5 to 9 years old [3,22,23,27]. Although the number of studies was limited, maternal disinhibited style and maternal BMI might be positively associated with EAH among girls only.. These findings show that familial characteristic might not have the same effect for boys and girls. 

Many studies found associations between eating behaviors promoting excessive energy intake and increased adiposity during childhood [85,86]. Although results were inconsistent during adolescence, our systematic review confirms the positive cross-sectional and longitudinal associations between EAH and adiposity during childhood, as also reported by Lansigan et al. in their 2015 systematic review [2]. Since obesity is so difficult to reverse, many experts argue that efforts should focus on prevention, more specifically during childhood [87,88]. Based on these evidences, stopping the development of unfavorable eating behaviors in early life, including EAH, could be a promising strategy for childhood obesity prevention programs. 

EAH can be observed in children as early as 3 years old [2], suggesting that the first 2 years of life, which have been found to be important for the development of eating behaviors and dietary patterns [89], may also be important in the development of EAH. Only few studies investigated associations between perinatal factors and EAH. Both studies assessing in utero exposure to maternal glucose intolerance found sex-specific associations with EAH during adolescence, and girls exposed in utero to some level of maternal glucose intolerance engaged in more EAH [47,48]. Breastfeeding practices (e.g., exclusivity, duration, breastfed vs. bottle-fed) may have an impact on eating regulation during childhood [90,91,92,93] and could play a part in the prevention of obesity [89,94,95]. Studies assessing breastfeeding practices and EAH were limited in number (n = 3). Reyes et al. reported a negative association between EAH and breastfeeding exposure for more than 6 months in 16–17 year old Chilean adolescents [46]. On the other hand, EAH was not associated with breastfeeding duration in two studies during early childhood [57,67]. Other than the age of EAH assessment, the different definitions of breastfeeding exposure used and the different socio-cultural characteristics between studied populations may explain the discrepancies. Studies focusing on the effect of the complementary feeding period on the development of EAH are also lacking. Only one study examined the association of EAH and a child’s age at complementary feeding introduction and found no association [57]. To better understand how EAH develops and evolves throughout childhood, there is a need for robust longitudinal studies spanning from early life through adolescence.

The number of studies examining the genetic influence on EAH during childhood has expanded in the past few years, but studies in adolescents are still missing. The three studies included in this review consistently showed positive associations between EAH and the rs9939609 polymorphism of the FTO gene [17,59,68]. This polymorphism has been associated with higher BMI and has been shown to increase the risk of obesity in adults and children from age 7 [96]. Additionally, one study reported that EAH was similar between full siblings, but not between half-siblings (same biological mother) [16]. Thus, both parents might play a role in the heritability of EAH. More studies are needed to identify how the genetic background might interact with familial and environment characteristics to influence the development of EAH, and studies in adolescence are needed. 

Studies examining potential associations between EAH and environmental characteristics are still missing. As previously discussed by Lansigan et al. [2], a better understanding of the possible influence of environmental factors (e.g., food environment, including school food policies, and food publicity exposure) on EAH is necessary to fully understand the development of this behavior.

Interventions to prevent the development of EAH during childhood might be beneficial to reduce excess weight gain or obesity during that time. Boutelle et al. conducted three pilot studies (randomized trials) among 8–12 year old children, aiming to modify eating behaviors, including EAH [97,98,99]. First, children’s EAH decreased after an 8-week food cue exposure treatment, while appetite awareness training did not have any effect on children’s EAH [98]. Combining both treatments (food cue exposure and appetite awareness training) into a 12-week intervention resulted in decreased EAH compared to a control group (no intervention) [97], while an attention modification program might influence EAH in children with obesity [99]. In a pilot study, Savage et al. tested a 4-week multicomponent family-based behavioural intervention, including a home supply of candies, parent shared decision making, child mindfulness and child attention control strategies; however, no effect on EAH was observed in this randomized trial [49]. Lee et al. conducted a feasibility study to evaluate a 12-hour intervention on healthy eating and physical activity in 3–5 years old children and found that children’s EAH increased post-intervention [100]. In a cluster randomized controlled study, preschools were assigned to an intervention aiming to increase children’s inhibitory control towards snack foods or a control lifestyle intervention, and children’s EAH was higher after the intervention for both groups [101]. Finally, Schyns et al. compared, in a randomized trial, the effect of a cue exposure intervention to a lifestyle intervention among adolescents with excess weight [29]. They found less EAH for the exposed, as well as for the non-exposed food items in the cue exposure condition [29]. Preliminary findings from pilot studies thus suggest potential benefits from food cue exposure training to reduce EAH in children and in adolescents with excess weight or obesity; yet studies had a short follow-up duration and small sample sizes, and further studies are needed.

There are limitations in the current literature on factors associated with EAH, hindering our ability to draw conclusions. Mainly, we observed a significant variability in the EAH laboratory protocol across studies. For example, the operationalized measure of EAH was reported mainly in kcals (or kilojoules), but also in grams of food, percentage of daily caloric needs, and more. While the original EAH protocol [1] included 10 snacks (sweet and salty) in the free access phase, some studies offered only sweet snacks [28,29,60,61,69] or fewer (≤4) snacks [17,51,52,65,68,80,83,100]. Other divergent factors related to the EAH protocol across studies include the free access phase setting (individual vs. in group), the time between the pre-load meal and the free access phase, and the pre-load meal type (e.g., buffet meal, standardized meal, usual lunch). The current evidence on EAH’s correlates is also limited by the small number of longitudinal studies, especially throughout or during adolescence. The most robust longitudinal analyses were conducted from a same cohort of white girls only, from 5 to 13 years old [3,22,23,24,25,27]. The lack of larger prospective cohort studies might be explained by the nature of the EAH laboratory protocol which requires extensive resources and is quite long to conduct (~40 min). In this context, the EAH-C questionnaire [4] presents a useful tool to standardize and facilitate the assessment of EAH. Nonetheless, even when using the EAH-C questionnaire, we noticed discrepancies in methods across studies [32,41,42,43,47]. Some studies also opted to use the EAH-P questionnaire, although parental report of child’s EAH has been shown to differ from children’s self-reported EAH [39]. We did not find significant differences in EAH correlates by assessment method, but only a few studies used the questionnaire. 

## 5. Conclusions

In summary, studies showed that EAH is likely to increase during childhood and persist through adolescence. EAH was associated with several individual and familial factors. Notably, studies showed that during childhood, EAH might be greater among boys, EAH could be linked to a genetic predisposition, and EAH is positively associated with adiposity. Familial characteristics associated with EAH included parental use of restrictive feeding practices among girls. Due to current limitations in the literature, more studies are needed to better understand determinants of EAH and to design interventions targeting high-risk groups. Interventions starting as early as possible and including familial components such as feeding practices might be beneficial to prevent EAH, but there is a need for further research.

## Figures and Tables

**Table 1:** The overall conclusions for individual factors associated with eating in the absence of hunger (EAH) in children and adolescents.

Individual Factors	In Childhood (≤12 Years Old)	In Adolescence (>12 Years Old)
Age	EAH seems to increase with age ^2,3^	EAH slightly increases ^1^ or remains stable ^2^ with age
Sex	EAH is observed among boys and girls and tends to be more pronounced in boys	EAH is observed among boys and girls, with no clear differences between sex
Adiposity	EAH is positively associated with adiposity ^2,3^ Birthweight may be positively associated with EAH for girls only ^1,2^	Findings are inconsistent
Exposure to breastfeeding	No association found for breastfeeding exposure ^2^	EAH might be less pronounced in adolescents who were breastfed for > 6 months ^1,2^
In utero exposure to maternal glucose intolerance	–	Girls exposed in utero to maternal glucose intolerance engage in more EAH ^2,4^ Findings are inconsistent for boys ^2,4^
Genetics	EAH may have a genetic component; associations were found between EAH and the polymorphism rs9939609 of the FTO gene and with the 16p11.2 genotype ^1,4^, but not with the rs1800497 polymorphism of the dopamine D2 receptor gene ^3^ Findings for the rs17782313 polymorphism of the melanocortin-4 receptor gene were inconsistent	–
Eating behaviors	EAH is positively associated with appetitive self-regulation ^2^ EAH might be positively associated with the relative reinforcement value for food in girls ^1^, and with children’s response to meal size ^1^, external and emotional eating ^1^ No associations were found for other eating behaviors including food and satiety responsiveness, slowness in eating ^1^, enjoyment of food, emotional overeating ^1^, restrained eating ^1^, negative assessment of eating, caloric compensation index and portion size assessment	Loss of control (LOC) and binge-eating in adolescents are associated with more EAH EAH might be positively associated with restrained eating in girls ^1^ EAH might be positively associated with emotional eating ^1^
Neurobehavioral measures	EAH might be positively associated with stress ^2^ Negative affect might be negatively associated with EAH ^1^ Findings for approach behavior are inconsistent EAH is not associated with child’s temperament ^2^ or IQ ^1^	Findings for emotional regulation are inconsistent Dispositional mindfulness might be negatively associated with EAH
Lifestyle habits	Screen time ^1^, physical activity ^1^ and sleep patterns ^1^ are not associated with EAH	Findings for sleep patterns are inconsistent EAH might be linked to eating habits (energy and sugar-sweetened beverages intake) ^4^
Sociocultural pressures and body image	–	EAH might be associated with body weight stigma-related measures
Emotional state (affect)	Children >5 years old might engage in more EAH in the context of a negative mood induction ^1^	A sad mood induction does not affect EAH ^3^
Brain activity measures	EAH is positively associated with neuronal activity in regions of the reward network of the brain	–
Other individual factors	EAH might be associated with appetite-regulating hormones ^1^ and be influenced by food publicity ^1^ Findings for pubertal status are inconsistent. EAH is not associated with race/ethnicity ^1^, child acculturation ^1^, complementary feeding introduction ^1^ and nutritive sucking ^1^	EAH is associated with pubertal status, but not with race/ethnicity ^1^

^1^ Evidence is limited to one study; ^2^ Evidence emerges in part or completely from longitudinal analysis; ^3^ Evidence is partially or completely issued from the same cohort; ^4^ Evidence is only issued from studies where EAH was assessed by questionnaire.

**Table 2:** The overall conclusions for familial factors associated with eating in the absence of hunger (EAH) in children and adolescents.

Familial Factors	In Childhood (≤12 Years Old)	In Adolescence (>12 Years Old)
Parental adiposity	Girls with heavier mothers seem to engage in more EAH over time ^2,3^ Maternal pre-pregnancy body weight might be positively associated with EAH in boys ^1,2^	EAH is not associated with maternal body mass index ^1,4^ Girls whose mother had a lower pre-pregnancy body weight tend to engage in more EAH ^1,2^
Parental eating style	Maternal disinhibited eating style is positively associated with daughter’s EAH ^2^ EAH is not associated with parental dietary restraint ^1^	Maternal binge eating and EAH might be associated with greater child’s EAH ^1^
Parental restrictive feeding practices	Parental use of restrictive feeding practices is associated with greater EAH, mostly in girls ^2,3^	Parental restrictive feeding practices might be positively associated with EAH ^1^
Other parental feeding practices	Inconsistent associations with using food as rewards and parental pressure to eat Children whose mother moderately allow them to serve themselves during meals seem to engage in more EAH ^1^ Monitoring was not associated with EAH	No direct associations between EAH and parental monitoring and concern was found ^1^
Parental demographics	EAH is not associated with household socioeconomic status, maternal education ^2^ or maternal age at delivery ^1,2^	–
Other familial factors	EAH might be inversely associated with household obesogenic food availability ^1^, but not home environment quality ^1^ EAH tends to be positively associated with maternal use of psychological control ^1^, but not firm control and acceptance ^1^	–

^1^ Evidence is limited to one study; ^2^ Evidence emerges in part or completely from longitudinal analysis; ^3^ Evidence is partially or completely issued from the same cohort; ^4^ Evidence only comes from studies using the EAH-C questionnaire.

## Data Availability

The data used for this review comes from published manuscripts which are all listed in the references.

## Supplementary Materials

The following supporting information can be downloaded at: https://www.mdpi.com/article/10.3390/nu14224715/s1, Table S1: Characteristics of included studies assessing eating in the absence of hunger (EAH) during childhood (≤12 years old) with the laboratory protocol; Table S2: Characteristics of included studies assessing eating in the absence of hunger (EAH) during adolescence (>12 years old) with the laboratory protocol; Table S3: Characteristics of included studies assessing eating in the absence of hunger (EAH) during childhood (≤12 years old) with the questionnaire; Table S4: Characteristics of included studies assessing eating in the absence of hunger (EAH) during adolescence (>12 years old) with the questionnaire; Table S5: Characteristics of included studies assessing eating in the absence of hunger (EAH) during childhood (≤12 years old) or adolescence (>12 years old) with the laboratory protocol and the questionnaire.

## Abbreviations

EAH	Eating in the absence of hunger
kcals	kilocalories
EAH-C	EAH questionnaire for children and adolescents
EAH-P	parent-report version of the questionnaire
PRISMA	Preferred Reporting Items for Systematic reviews and Meta-Analyses
U.S.	United States
BMI	body mass index
GDM	gestational diabetes
CEBQ	Children Eating Behavior Questionnaire
DEBQ	Dutch Eating Behavior Questionnaire
LOC	loss of control
