# Supplementary material for: Factors Associated with Eating in the Absence of Hunger among Children and Adolescents: A Systematic Review
Source: Nutrients. 2022 Nov 9;14(22):4715. doi: 10.3390/nu14224715 (PMC9699171; doi:10.3390/nu14224715)
Supplement: Supplementary file 1 [file nutrients-14-04715-s001.zip › nutrients-1960479-supplementary.pdf]

Search strategy

We included all search terms synonymous to EAH; eating without hunger, eating when not hungry, eating when satiated, eating beyond (or [past]) satiety (or [satiation]), eating when full, eating beyond (or [past]) fullness, eating after a meal, eating after a snack, satiety responsiveness or response to satiation. Search terms related to "hedonic eating" such as hedonic (eating, snack, snacking, hunger) were also included since eating for pleasure can be associated with eating without hunger.

Table S1. Characteristics of included studies assessing eating in the absence of hunger (EAH) during childhood (≤ 12 years old) with the laboratory protocol.

Authors, publication date	Sample size and Characteristics	EAH assessment method	Child measures	Familial measures	Associations with EAH	Funding sources	Conflicts of interest	Quality assessment[1]
**Cross-sectional studies**								
Cutting et al. 1999	**n**=75<br>**Age range:** 3-6 yrs<br>**Mean age:** 4.95 ± 0,98 yrs<br>**Sex:** 47% female<br>**Race/Ethnicity:** from predominantly white families	**Setting:** Not reported<br>**Location:** PA, United States<br>**Pre-load:** Usual lunch<br>**Free access:** 10 palatable snacks (10 min)<br>**Operationalized EAH measure:** kJ	Adiposity (weight-for-height ratio)<br>Sex	Paternal and maternal eating behavior (disinhibition and restraint)<br>Paternal and maternal weight status	Positive association between EAH and adiposity, in girls only<br>Positive association of EAH and maternal disinhibition, in girls only<br>Positive association between EAH and maternal BMI, via maternal disinhibition	NIH	Not reported	***
Fisher & Birch 1999	**n**=56<br>**Age range:** 4-6 yrs<br>**Mean age:** 5.0 ± 0.1 yrs<br>**Sex:** 43% female<br>**Race/Ethnicity:** 86% Caucasian, 9% Asian, 4% African American, 1% Hispanic.	**Setting:** Not reported<br>**Location:** PA, United States<br>**Pre-load:** Usual lunch<br>**Free access:** (right after)**:** 10 palatable snacks (10 min)<br>**Operationalized EAH measure:** kcal	Perception of maternal restriction to palatable foods present in the EAH procedure<br>Sex	Maternal child-feeding practices (restricting access to palatable food present in the EAH procedure)	Positive association between EAH and maternal restriction of palatable foods and perception of maternal restriction, in girls only	Not reported	Not reported	****
Birch & Fisher 2000	**n**=156[2]<br>**Age range:** 4.6-6.4 yrs<br>**Mean age:** 5.4 ± 0.02 yrs	**Setting:** Laboratory<br>**Location:** IL, United States<br>**Pre-load:** Standardized lunch	None	Maternal and paternal child-feeding practices (restraint and disinhibition)	Positive association between EAH and maternal restriction of access to palatable foods	NIH<br>National Dairy Council	Not reported	***

	**Sex:** 100% female<br>**Race/Ethnicity:** 100% white	**Free access:**10 palatable snacks (10 min)<br>**Operationalized EAH measure:** kJ		and maternal restriction of access to palatable food				
Fisher & Birch 2000	**n**=158[2]<br>**Age range:** 4.6-6.4 yrs<br>**Mean age:** Not reported<br>**Sex:** 100% female<br>**Race/Ethnicity:** 100% white	**Setting:** Laboratory (presumed)<br>**Location:** IL, United States<br>**Pre-load:** Standardized lunch<br>**Free access:**10 palatable snacks (10 min)<br>**Operationalized EAH measure:** kcal	Negative self-evaluation of number of snacks consumed and negative emotion about eating<br>Perception of parental restriction	Parental child-feeding practices (restricting access to palatable food present in the EAH procedure)	Positive association between EAH and parental restriction of palatable foods	Not reported	Not reported	***
Wardle et al. 2001	**n**=428 twins<br>**Age range:** 4-5 yrs<br>**Mean age:** 4.43 ± 0.34 yrs<br>**Sex:** 52% female<br>**Race/Ethnicity:** Not reported	**Setting:** Home<br>**Location:** United Kingdom<br>**Pre-load:** Usual meal (presumed)<br>**Free access** (1 hour later)**:** 3 variety of biscuit<br>**Operationalized EAH measure:** g of biscuits	None	Parental weight status: overweight/obese, normal weight/lean	No significant associations with EAH	Biotechnology and Biological Sciences Research Council	Not reported	**
Fisher et al. 2007	**n**=801 from 301 families<br>**Age range:** 5-18 yrs<br>**Mean age:** 11.7 yrs<br>**Sex:** 48% female<br>**Race/Ethnicity:** 100% white	**Setting:** Laboratory<br>**Location:** TX, United states<br>**Pre-load:** Standardized dinner (20 min)<br>**Free access:** 10 palatable and	Adiposity (BMI z-score adjusted for age and sex ≥ 95 percentile)<br>Age<br>Child<br>Acculturation<br>Sex	Household food security	Greater EAH for boys than girls<br>EAH increased with age until 13 yrs<br>Positive association between EAH and adiposity<br>EAH had a heritability component	NIH, NIDDK<br>U.S Department of Agriculture<br>Baylor College of Medicine	Not reported	***

		healthy snacks (10-15 min)<br>**Operationalized EAH measure:** kcal and % total energy expenditure	Fasting hormonal level (ghrelin, amylin, leptin, and insulin)		Positive association between EAH and fasting leptin and insulin levels			
Moens & Braet 2007	**n**=25<br>**Age range:** 8-12 yrs<br>**Mean age:** 10.24 ± 1.14 yrs<br>**Sex:** 40% female<br>**Race/Ethnicity:** 56% white, 40% black, 4% Asian	**Setting:** Home, led by experimenter<br>**Location:** Belgium (presumed)<br>**Pre-load:** Typical family dinner<br>**Free access:** 10 palatable snacks (20 min), while filmed<br>**Operationalized EAH measure:** g and number of snacks	Adiposity (BMI z-score adjusted for age and sex ≥ 95 percentile)<br>Age<br>Eating behavior (external eating, emotional eating and restraint eating)<br>Sex	Index of social position<br>Maternal BMI<br>Parental child-feeding practices (restriction, pressure to eat and monitoring)	Negative association between EAH and adiposity, in girls only<br>Positive associations between EAH and external eating and emotional eating	Not reported	Not reported	***
Hill et al. 2008	**Study 1**<br>**n**=348<br>**Age range:** 7-9 yrs<br>**Mean age:** 8.28± 0.64 yrs<br>**Sex:** 51% female<br>**Race/Ethnicity:** 45% white, 51% non-white, 4% unknown	**Setting:** School<br>**Location:** United Kingdom<br>**Pre-load:** Usual lunch<br>**Free access** (20 min later)**:** 7 sweet snacks, in groups (10 min)<br>**Operationalized EAH measure:** g	Adiposity (BMI z-score adjusted for age and sex)<br>Sex	None	Greater EAH among boys compared to girls, in both studies<br>Positive association between EAH and adiposity, among boys only, in both studies	Cancer Research UK	Not reported	***
	**Study 2**<br>**n**=316 twins<br>**Age range:** 9-12 yrs	**Setting:** Home<br>**Location:** United Kingdom<br>**Pre-load:** Standardized meal						

	**Mean age:** 11.7± 0.54 yrs<br>**Sex:** 61% female<br>**Race/Ethnicity:** 93% white, 7% non-white	**Free access** (10 min later): 8 sweet snacks with their twins (10 min)<br>**Operationalized EAH measure:** g						
Wardle et al.<br>2009	**n**=131<br>**Age range:** 4-5 yrs<br>**Mean age:** 4.4 yrs<br>**Sex:** 68% female<br>**Race/Ethnicity:** Not reported	**Setting:** Home<br>**Location:** United Kingdom<br>**Pre-load:** Meal prepared by mothers<br>**Free access (1 hour later):** 3 variety of biscuits (10 min)<br>**Operationalized EAH measure:** g	Rs9939609 genotype (TT, AT, AA)	None	Less EAH among children with a TT genotype compared to children with an AT or AA genotype	Biotechnology and Biologial Sciences Research Council	None	**
Blisset et al. 2010	**n**=25<br>**Age range:** 3-5 yrs<br>**Mean age:** 4.1 yrs<br>**Sex:** 54% female<br>**Race/Ethnicity:** 88% white	**Setting:** Laboratory<br>**Location:** United Kingdom<br>**Pre-load:** Standardized lunch<br>**Free access:** 6 palatable and healthy snacks (4 min)<br>**Operationalized EAH measure:** kcal	Experimental group with negative mood induction or control group	Maternal child-feeding practices (emotional regulation, pressure to eat, using food as a reward, restriction for health and for weight)	Positive association between EAH for cookies and maternal use of food to regulate their child's emotion, independently of mood condition<br>Positive association between EAH for chocolate and maternal use of food to regulate their child's emotion in the experimental group, compared to control group<br>Greater EAH of breadsticks among children in the experimental group compared to control group	Authors' research funds	None	**
Kral et al. 2012	**n**=47 weight discordant siblings	**Setting:** Laboratory<br>**Location:** PA, United States	Adiposity (BMI z-score adjusted	None	Within pair resemblances in EAH in full siblings, but not in half-siblings	Mentored Research Scientist Development	Financial interests with the	**

	**Age range:** 5-12 yrs<br>**Mean age:** 8.9 ± 2.3 yrs<br>**Sex:** 53% female<br>**Race/Ethnicity:** 68% African American, 9% white, 19 % more than one race, 4% unknown; (20% Hispanic, 54% not Hispanic, 26% unknown)	**Pre-load:** Pudding + standardized dinner<br>**Free access** (15 min later)**:** 6 palatable snacks (15 min)<br>**Operationalized EAH measure:** kcal	for age and sex ≥ 85 percentile)<br>Age<br>Sex<br>Caloric compensation index<br>Sibling differences in fat-free mass, % body fat and BMI z-score		Greater EAH among siblings with excess weight/obesity vs. normal weight siblings<br>Age and sex interactions: greater EAH among older siblings with excess weight/obesity vs. older and younger siblings with normal weight and higher EAH among boys with excess weight/obesity vs. girls with excess weight/obesity or normal weight<br>Positive association between siblings' differences in fat-free mass and siblings' differences in EAH	Award from NIDDK of the NIH	Frontiers Foundation; Vivus Inc; Kraft Foods;<br>University of Wisconsin; University of Arizona; Paul, Weiss, Wharton &<br>Garrison LLP; and Sage Publications	
Francis et al.<br>2013	**n**=43<br>**Age range:** 5-9 yrs<br>**Mean age:** 6.6 ± 1.4 yrs<br>**Sex:** 39% female<br>**Race/Ethnicity:** 68% white, 16% black and 16% other	**Setting:** Laboratory<br>**Location:** PA, United States<br>**Pre-load:** Standardized lunch<br>**Free access** (20 min later)**:** 10 palatable snacks (10 min)<br>**Operationalized EAH measure:** kcal	Age<br>Cortisol release when exposed to a stressor<br>Parental report of child's impulsivity	None	Positive association between EAH and cortisol's release in response to stress (immediately post-stress and after the stressor has passed), in older children only (8-9 yrs)	Children, Youth and Families Consortium at The Pennsylvania State University<br>NIH	One author is the founder and Chief Scientific and Strategy advisor at Salimetrics LLC (State College, PA)	**
Harris et al.<br>2014	**n**=37<br>**Age range:** 3.8-4.5 yrs<br>**Mean age:** 4.3 yrs<br>**Sex:** 57% female<br>**Race/Ethnicity:** Not reported	**Setting:** Home, led by mother<br>**Location:** Australia<br>**Pre-load:** Semi-standardized lunch (30 min)	Sex	Maternal child-feeding practices (Pressure to eat, restriction and monitoring)	Positive association between EAH and maternal use of pressure to eating, among boys only	The Australian National Health and Medical Research Council<br>H. J. Heinz (post-doctoral fellowship)	None	**

		**Free access** (15 min later)**:** 5 palatable snacks (15 min)<br>**Operationalized EAH measure:** kJ				Meat & Livestock Australia<br>Department of Health South Australia<br>Food Standards Australia New Zealand<br>Queensland University of Technology		
Ho-Urriola et al.<br>2014	**n**=10^{2}<br>**Age range:** 6-12 yrs<br>**Mean age:** 10.4 ± 2.2 yrs<br>**Sex:** 47% female<br>**Race/Ethnicity:** Not reported	**Setting:** Laboratory<br>**Location:** Chile<br>**Pre-load:** Standardized lunch<br>**Free access:** 5 sweet snacks<br>**Operationalized EAH measure:** kcal and g	MC4R rs17782313 genotype (C-allele carriers and non-carriers)	None	No significant associations with EAH	Chilean grants FONDECYT	None	**
Leung et al.<br>2014	**n**=380 from low-income families<br>**Age range:** 3-4 yrs<br>**Mean age:** 4.10 ± 0.54 yrs<br>**Sex:** 50% female<br>**Ethnicity:** 56% non-Hispanic white, 16% non-Hispanic black, 17% bon-Hispanic multiracial and 11% Hispanic.	**Setting:** Preschool<br>**Location:** MI, United States<br>**Pre-load:** Non-standardized breakfast<br>**Free access:** 6 palatable<br>snacks (10 min)<br>**Operationalized EAH measure:** kcal	Temperament (surgency level, effortful control, and negative affectivity)	Home environment quality score	Positive association between EAH and surgency<br>More EAH associated with less negative affectivity	Intramural Research Program from the NICHD	None	***

Boutelle et al.<br>2015	**n**=23 (10 children with obesity matched with 13 children with normal weight)<br>**Age range:** 8-12 yrs<br>**Mean age:** 10.2 ± 0.3 yrs<br>**Sex:** 43% female<br>**Race/Ethnicity:** Not reported	**Setting:** Laboratory<br>**Location:** CA, United States<br>**Pre-load:** Standardized pizza dinner<br>**Free access** (10 min later)**:** 11 palatable snacks (10 min)<br>**Operationalized EAH measure:** % of daily caloric needs	Adiposity (BMI z-score adjusted for age and sex ≥ 95 percentile)<br>Brain activation to water and sucrose (amygdala, gyrus, putamen, insula, and striatum)	None	No significant associations with EAH	NIH<br>Price Foundation Davis/Wismer Foundation	None	**
Hugues et al. 2015	**n**=18, from low-income families<br>**Age range:** 4-5 yrs<br>**Mean age:** 4.9 ± 0.4 yrs<br>**Sex:** 83% female<br>**Race/Ethnicity:** 100% Hispanic	**Setting:** Laboratory<br>**Location:** TX, United States<br>**Pre-load:** Standardized meal<br>**Free access** (immediately after)**:** 7 palatable snacks (10 min)<br>**Operationalized EAH measure:** kcal	Adiposity (BMI z-score adjusted for age and sex)<br>Age<br>Appetitive self-regulation[3] (delay of gratification task)<br>Eating behaviors (satiety + food responsiveness)<br>Non-appetitive regulation (inhibitory control, cognitive flexibility, effortful control, and emotion regulation)<br>Sex	None	Positive correlation between EAH and appetitive self-regulation<br>Positive associations between EAH and adiposity and age<br>Greater EAH among boys compared to girls	NICHD<br>US Department of Agriculture/Agricultural Research Service	Not reported	***
Remy et al.<br>2015	**n**=221<br>**Age range:** 3-6 yrs	**Setting:** School<br>**Location:** France	Adiposity (BMI z-score adjusted for age and sex,	Maternal child-feeding practices (using	Greater EAH among boys compared to girls	European Community's Seventh	None	***

	**Mean age:** 4.50 ± 0.06 yrs<br>**Sex:** 46% female<br>**Race/Ethnicity:** Not reported	**Pre-load:** Standardized lunch<br>**Free access** (10 min later)**:** 10 palatable snacks (10 min) in group<br>**Operationalized EAH measure:** % of pre-load energy intake and kcal	waist circumference)<br>Age<br>Caloric compensation index<br>Sex	food as a reward, emotional regulation, pressure to eat, restriction for weight, monitoring, and child control)	Positive association between EAH (kcals) and BMI z-score<br>Positive association between EAH (% of pre-load) and maternal use of food as a reward	Framework Program<br>Regional Council of Burgundy<br>European Funding for Regional Economical Development<br>Foundation Louis Bonduelle		
Power et al.<br>2016	**n**=187, from low-income families<br>**Age range:** 4-5 yrs<br>**Mean age:** 4.8 ± 0.4 yrs<br>**Sex:** 48% female<br>**Race/Ethnicity:** 100% Hispanic	**Setting:** Laboratory<br>**Location:** TX, United States<br>**Pre-load:** Standardized meal<br>**Free access** (immediately after)**:** 7 palatable snacks (10 min)<br>**Operationalized EAH measure:** kcal	Adiposity (BMI z-score adjusted for age and sex ≥ 85 percentile)<br>Age<br>Appetitive self-regulation[3] (delay of gratification task)	None	Positive associations between EAH and age, adiposity, and appetitive self-regulation	NICHD<br>U.S Department of Agriculture/Agricultural Research Service	Not reported	***
Liang et al.<br>2016	**n**=117 children with excess weight or obesity<br>**Age range:** 7-12 yrs<br>**Mean age:** 10.40 ± 1.40 yrs<br>**Sex:** 53% female<br>**Race/Ethnicity:** 54% Caucasian, 20% mixed, 14% African	**Setting:** Laboratory<br>**Location:** MN, United States<br>**Pre-load:** Standardized dinner<br>**Free access** (10 min later)**:** 11 palatable snacks (10 min)<br>**Operationalized EAH measure::** % of daily caloric needs (for total EAH and sweet snacks only)	Adiposity (BMI)<br>Age<br>Sex	Child's report of parental (maternal) behavior (acceptance, psychological control, and firm control)<br>Parental child-feeding practices (restriction, pressure to eat, and monitoring)	Greater total EAH among girls compared to boys<br>Positive association between EAH from sweet snacks and parental monitoring<br>Positive association between total EAH and parental use of psychological control<br>Positive associations between EAH of Hersey chocolate bars and all parental child-feeding practices and	University of Minnesota Faculty Development	Not reported	***

	American, 12% other			Parental BMI	maternal psychological control			
Silva Garcia et al. 2016	**n**=186<br>**Age range:** 4-5 yrs<br>**Mean age:** 4.34 ± 0.48 yrs<br>**Sex:** 48% female<br>**Race/Ethnicity:** 100% Hispanic	**Setting:** Laboratory<br>**Location:** TX, United States<br>**Pre-load:** Standardized meal<br>**Free access:** 7 palatable snacks (10 min)<br>**Operationalized EAH measure:** kcal	Adiposity (BMI z-score adjusted for age and sex ≥85 percentile)<br>Age<br>Eating behavior (food responsiveness, satiety responsiveness, and emotional overeating)<br>Sex	Observed maternal serving practices (allowing the child choice, allowing the child to serve, and lack of restriction)	Positive associations of EAH with age and adiposity<br>Greater EAH among boys compared to girls<br>Higher EAH intake from children whose mother moderately allow them to serve themselves	NICHD<br>U.S. Department of Agriculture/Agricultural Research Service	Not reported	****
Gearhardt et al. 2017	**n**=230<br>**Age range:** 7-10 yrs<br>**Mean age:** 7.84 ± 0.66 yrs<br>**Sex:** 49% female<br>**Race/Ethnicity:** 54% non-Hispanic white, 15% non-Hispanic black, 19% biracial non-Hispanic, 10% Hispanic, 1% non-Hispanic other	**Setting:** Laboratory<br>**Location:** MI, United States<br>**Pre-load:** Standardized meal<br>**Free access** (immediately after)**:** 6 sweet deserts (5 min)<br>**Operationalized EAH measure:** kcal	Adiposity (BMI z-score adjusted for age and sex ≥ 85 percentile)<br>Appetitive self-regulation (reinforcement value for food, and delay of gratification)	None	Positive association between EAH and adiposity, for girls only<br>Positive association between EAH and reinforcement value of food, for girls only	NIH	None	***
Gilbert-Diamond et al. 2017	**n**=172<br>**Age range:** 9-10 yrs	**Setting:** Laboratory<br>**Location:** NH, United States	Adiposity (BMI z-score adjusted for age and sex ≥ 85 percentile)	Household income<br>Maternal education	Greater EAH among boys compared to girls<br>Positive association between EAH and adiposity	NICHD<br>National Institute of Drug Addiction	None	****

	**Mean age:** 9.9 ± 0.6 yrs<br>**Sex:** 43% female<br>**Race/Ethnicity:** 86% white, 14% non-white, 97% non-Hispanic, 3% Hispanic	**Pre-load:** Self-selected standardized lunch<br>**Free access** (20 min later)**:** 4 palatable snacks while watching a 34-minute show<br>**Operationalized EAH measure:** kcal	Age<br>Experimental group (food advertisement of gummy candy) or control group<br>Physical activity<br>Rs9939609 genotype (TT, AT, AA)<br>Screen time<br>Sex	Parental child-feeding practices (restriction)	Positive association between EAH and parental use of food restriction<br>Positive effect of the experimental condition on children's EAH, for the snack advertised only<br>Linear increased of EAH of the snack advertised from TT genotype through AA genotype, in the experimental group	National Cancer Institute<br>National Institute of Alcohol Abuse and Alcoholism<br>NIH<br>National Institute of Environmental Health Sciences Environmental Protection Agency		
Mestre et al.<br>2017	**n**=25<br>**Age range:** 8-12 yrs<br>**Mean age:** 10.24 ± 1.14 yrs<br>**Sex:** 40% female<br>**Race/Ethnicity:** 56% white, 40% black, 4% Asian	**Setting:** Laboratory<br>**Location:** CA, United States<br>**Pre-load:** Standardized pizza dinner<br>**Free access** (10 min later)**:** 11 palatable snacks (10 min)<br>**Operationalized EAH measure:** % of daily caloric needs	Adiposity (BMI z-score adjusted for age and sex ≥ 95 percentile)<br>Bilateral hippocampus response to taste test<br>Hippocampus volume	None	Positive association between EAH and activation following the taste test, in the ventral left hippocampus	Department of Pediatrics, University of California San Diego<br>NIH<br>Price Foundation<br>Davis/Wismer Foundation	None	**
Adise et al.<br>2018	**n**=46<br>**Age range:** 7-11 yrs<br>**Mean age:** 9.0 ± 1.3 yrs<br>**Sex:** 54% female<br>**Race/Ethnicity:** 93% white, 5% black, 2% Asian	**Setting:** Laboratory<br>**Location:** PA, United States<br>**Pre-load:** Standardized lunch or dinner (30 min)<br>**Free access** (20 min later)**:** 10 palatable snacks (15 min)	Adiposity (BMI z-score adjusted for age and sex ≥ 85 percentile)<br>BOLD responses to anticipating food compared to money<br>Pubertal Status<br>Sex	None	Higher EAH among boys compared to girls<br>Positive association between BOLD response in the dorsolateral prefrontal cortex for winning food compared to money and EAH at two different sites (R1 & R2)	Childhood Obesity Prevention Training Grant<br>The Pennsylvania State University's Social, Life & Engineering Sciences Imaging Center	None	**

		**Operationalized EAH measure:** kcal						
Boots et al. 2018	**n**=213<br>**Age range:** 3-5 yrs<br>**Mean age:** 4.8 yrs<br>**Sex:** 51% female<br>**Race/Ethnicity:** Not reported	**Setting:** Preschool classroom<br>**Location:** Australia<br>**Pre-load:** Buffet lunch<br>**Free access** (20 min later)**:** 3 palatable snacks, in group (10 min)<br>**Operationalized EAH measure:** kcal	Sex	Maternal child-feeding practices (restriction, pressure to eat and covert control)	Boys engaged in more EAH compared to girls<br>Association between maternal use of pressure to eat and less EAH, for boys only<br>Association between maternal use of restriction and more EAH, in girls only	Not reported	Not reported	***
Boone-Heinonen et al. 2019	**ABC preschool**<br>**n**=307<br>**Age range:** 3-5 yrs<br>**Sex:** 69% female	**Setting:** Head start preschool<br>**Pre-load:** Breakfast<br>**Free access:** 6 palatable snacks (10 min)	Birth weight z-score<br>Eating behavior (food responsiveness, enjoyment of food and satiety responsiveness)	Gestational weight gain<br>Pre-pregnancy BMI	Positive association between higher and lower birthweight z-score and EAH (from sweet snacks and total), among girls only<br>Positive correlation between EAH (from sweet snacks and total) and food responsiveness	NICHD<br>NIDDK	None	***
	**ABC Toddlers**<br>**n**=147<br>**Age:** 33 months<br>**Sex:** 32% female	**Setting:** Home<br>**Pre-load:** Typical lunch<br>**Free access:** 7 palatable snacks (10 min)						
	**N total**=454<br>**Mean age:** 3.8 yrs<br>**Sex:** 49% female<br>**Race/Ethnicity:** 54% non-Hispanic white, 19% non-Hispanic black,	**Location:** MI, United States<br>**Operationalized EAH measure:** kcal						

	16% non-Hispanic biracial or other, 10% Hispanic							
Fogel et al. 2019	**n**=255<br>**Age:** 6 yrs<br>**Sex:** 50% female<br>**Race/Ethnicity:** 64% Chinese, 16% Indian, 20% Malay	**Setting:** Laboratory<br>**Location:** Singapore<br>**Pre-load:** Vegetarian fried rice self-served (20-30 min)<br>**Free access** (20 min after)**:** 4 palatable snacks (5 min)<br>**Operationalized EAH measure:** kcal	Behavior during Stop Signal Task (restless vs typical)<br>Temperament (inhibitory control (stop signal task))<br>Sex	None	Greater EAH among boys compared to girls<br>Greater EAH among children who had a restless behavior compared to the ones who had a typical behavior during the Stop Signal Task	Singapore National Research Foundation<br>Singapore Institute for Clinical Sciences A*STAR and Nestec<br>National Institute for Health Research Southampton Biomedical Research Centre<br>European Union's Seventh Framework Programme, project Early Nutrition	Speaker at conference sponsored by companies selling nutritional products.<br>Part of a consortium with funding from Abbott Nutrition, Nestec and Danone.<br>Employee of Nestec SA (Nestlé Research Center)	***
Miller et al. 2019	**n**=223 from low-income families<br>**Age range:** 7-10.5 yrs<br>**Mean age:** 7.8 ± 0.7 yrs<br>**Sex:** 50% female<br>**Race/Ethnicity:** 55% non-Hispanic white, 20% biracial, 15%	**Setting:** Preschool<br>**Location:** MI, United States<br>**Pre-load:** Standardized dinner<br>**Free access:** 6 sweet deserts (5 min)<br>**Operationalized EAH measure:** kcal	Adiposity (BMI z-score adjusted for age and sex ≥ 85 percentile)<br>Pubertal development<br>Sex<br>Stress response to a stress induction protocol (observed anxiety	None	Positive associations between EAH and pubertal development and adiposity<br>Positive association between EAH and observed anxiety, in children with excess weight only	NIH	None	**

	black, 10% Hispanic, 1% other		and self-reported distress)					
Shapiro et al. 2019	**n**=18 **Age range:** 4-6 yrs **Mean age:** 5.8 ± 0.5 yrs **Sex:** 61% female **Race/Ethnicity:** 61% non-Hispanic white, 17% non-Hispanic black, 22% Hispanic	**Setting:** Laboratory **Location:** CO, United states **Pre-load:** Standardized lunch **Free access:** 7 palatable snacks (10 min) **Operationalized EAH measure:** kcal	Intrinsic neuronal activity and connectivity (salience, default mode, executive control, and reward networks)	None	Positive associations between EAH and the rewards network neuronal activity and communication within the reward network Negative associations between EAH and reward network connectivity with the executive control network and the default mode networks	NIH NIDDK	None	**
Fogel et al. 2020	**n**=292 **Age:** 6 yrs **Sex:** 49% female **Race/Ethnicity:** 57% Chinese, 17% Indian, 26% Malay	**Setting:** Laboratory **Location:** Singapore **Pre-load:** Vegetarian fried rice self-served (20-30 min) **Free access** (20 min after)**:** 4 palatable snacks (5 min) **Operationalized EAH measure:** kcal	Child's overweight risk score (based on maternal pre-pregnancy overweight/obesity, paternal overweight/obesity at 24 months, maternal excessive gestational weight gain, high fasting plasma glucose during pregnancy, short breastfeeding duration and early introduction of solid foods)	None	No significant associations with EAH	Singapore National Research Foundation Singapore Institute for Clinical Sciences, Agency for Science Technology and Research UK Medical Research Council NIH NIH Research Southampton Biomedical Research Centre European Union's Erasmus+Capacit	Speaker at conference sponsored by companies selling nutritional products. Part of a consortium with funding from Abbott Nutrition, Nestec and Danone. Part of the scientific advisory council for Kerry Taste	***

						y-Building ENeASA Project and Seventh Framework Programme, project Early Nutrition	and Nutrition	
Kral et al. 2020	**n**=212<br>**Age range:** 7-9 yrs<br>**Mean age:** 8.4 ± 0.79 yrs<br>**Sex:** 59% female<br>**Race/Ethnicity:** 69% black or African American, 10% white, 18% more than one race, 3% other or unknown; 70% non-Hispanic, 8% Hispanic, 4% unknown	**Setting:** Laboratory<br>**Location:** PA, United States<br>**Pre-load:** Standardized lunch and dinner<br>**Free access** (15 min after)**:** 6 palatable snacks (15 min)<br>**Operationalized EAH measure:** kcal	Adiposity (BMI z-score adjusted for age and sex ≥ 85 percentile)<br>Race/ethnicity<br>Sex	Familial risk for obesity (based on maternal BMI)<br>Household income<br>Household food security<br>Obesogenic household food availability	Greater EAH among children with obesity and a higher risk for obesity compared to children with a normal weight and a lower or a higher risk for obesity<br>Greater EAH among boys compared to girls<br>Association between less EAH and higher obesogenic household food availability	NIDDK	None	***
Warkentin et al.<br>2020	**n**=70<br>**Age range:** 3-5 yrs<br>**Mean age:** 5.0 ± 0.5 yrs<br>**Sex:** 47% female<br>**Race/Ethnicity:** 48% white British, 6% white European, 5% black African, 6% other	**Setting:** School<br>**Location:** United Kingdom<br>**Pre-load:** Standardized lunch<br>**Free access** (30 min after)**:** 15 mini-cookies in group (10 min)<br>**Operationalized EAH measure:** kcal	None	Maternal child-feeding practices (restriction, monitoring, pressure to eat and prompting to eat)	Association between smaller EAH and higher use of parental monitoring	NIH<br>NIDDK<br>Cancer Research UK<br>Medical Research Council	None	**

**Prospective cohort studies**								
Fisher & Birch 2002	**n**=181<br>**Age range:** 5 yrs<br>**Sex:** 100% female<br>**Race/Ethnicity:** 100% non-Hispanic white<br>Baseline, 7yrs	**Setting:** Laboratory<br>**Location:** PA, United States<br>**Pre-load:** Standardized lunch<br>**Free access:** 10 palatable snacks (10 min)<br>**Operationalized EAH measure:** kcal	Adiposity (BMI z-score adjusted for age and sex ≥ 85 percentile)<br>Age	Parental child-feeding practices (restriction) at 5yrs	EAH was stable between 5 yrs and 7 yrs<br>Higher odds of EAH at 7 yrs for girls who were overweight at 5 yrs<br>Positive association between EAH at 7 yrs and parental use of restrictive feeding practices at 5 yrs	NIH<br>National Dairy Council	Not reported	***
Birch et al. 2003	**n**=140<br>**Age range:** 5 yrs<br>**Sex:** 100% female<br>**Race/Ethnicity:** 100% non-Hispanic white<br>Baseline, 7 yrs and 9 yrs	**Setting:** Laboratory<br>**Location:** PA, United States<br>**Pre-load:** Standardized lunch<br>**Free access** (immediately after)**:** 10 palatable snacks (10 min)<br>**Operationalized EAH measure:** kcal	Adiposity (BMI adjusted for age and sex z-score ≥ 75th percentile)<br>Age	Maternal child-feeding practices (restriction) at 5yrs	EAH increased from 5 yrs to 7 yrs and 9yrs<br>Higher odds of EAH at 7 yrs and 9 yrs for girls whose mother had a higher use of restrictive feeding practices at 5 yrs<br>Interaction with weight status at 9 yrs: highest EAH with the most important increase in the 4 yrs period among overweight daughters of mothers with a higher use of restrictive feeding practices	National Dairy council<br>NIH	Not reported	***
Shunk & Birch 2004	**n**=153<br>**Age:** 5 yrs<br>**Sex:** 100% female<br>**Race/Ethnicity:** 100% non-Hispanic white	**Setting:** Laboratory<br>**Location:** PA, United States<br>**Pre-load:** Self-selected lunch<br>**Free access:** 10 palatable snacks (10 min)	Adiposity (BMI z-score adjusted for age and sex ≥ 85 percentile) at 5yrs<br>Age	None	Positive prospective associations between EAH at 7 yrs and 9 yrs and adiposity at 5 yrs<br>Positive association between EAH increase over 4 yrs and adiposity	NIH<br>National Dairy Council<br>General Clinical Research Center of the Pennsylvania State University	Not reported	***

	Baseline, 7 yrs and 9 yrs	**Operationalized EAH measure:** kcal						
Faith et al. 2005	**n**=53<br>**Age:** 5 yrs<br>**Sex:** 51% female<br>**Race/Ethnicity:** 100% white	**Setting:** Laboratory<br>**Location:** PA, United States<br>**Pre-load:** Standardized dinner (20 min)<br>**Free access:** 11 palatable snacks (10 min)<br>**Operationalized EAH measure:** kcal	Nutritive sucking at 3 months<br>Sex	Mother pre-pregnancy weight<br>Parental child-feeding practices (limitation in snack food frequency)	Positive association between EAH and mother's pre-pregnancy weight among boys	NIH<br>Clinical Research Center<br>Nutrition Center of the Children's Hospital of Philadelphia	Not reported	***
Francis et Birch 2005	**n**=171<br>**Age:** 5 yrs<br>**Sex:** 100% female<br>**Race/Ethnicity:** 100% non-Hispanic white<br>Baseline, 7yrs and 9 yrs	**Setting:** Laboratory<br>**Location:** PA, United States<br>**Pre-load:** Standardized lunch<br>**Free access:** 10 palatable snacks (10 min)<br>**Operationalized EAH measure:** kcal	Adiposity (BMI z-score adjusted for age and sex ≥ 85 percentile)<br>Age	Maternal child-feeding practices (restriction)<br>Maternal weight status (overweight vs normal weight)	Greater EAH at 5 yrs among girls whose mother had a normal weight compared to the ones whose mother had an excess weight.<br>Greater EAH increase from 5-9 yrs among girls whose mother had an excess weight compared to daughters of mothers with normal weight<br>Positive associations between EAH across time points and baseline BMI z-score and BMI z-score increases, for daughters of mothers with excess weight only<br>Positive associations between EAH at each time points and maternal use of restriction at baseline, for daughters of mothers with excess weight only	NIH	Not reported	***

Francis et al.<br>2007	**n**=197<br>**Age:** 5 yrs<br>**Sex:** 100% female<br>**Race/Ethnicity:** 100% non-Hispanic white<br>Baseline, 7yrs (n=192), 9yrs (n=183), 11yrs (n=177) and 13 yrs (n=168)	**Setting:** Laboratory<br>**Location:** PA, United States<br>**Pre-load:** Not reported<br>**Free access:** 10 palatable snacks (a few minutes, presumably ~10 min)<br>**Operationalized EAH measure:** kcal	Age	Paternal and maternal weight status (overweight vs normal weight)<br>Paternal and maternal disinhibited eating style at baseline	Positive correlation between EAH at 9 yrs, 11 yrs and 13 yrs and maternal (not paternal) disinhibited eating style at baseline<br>EAH increased between baseline and 13 yrs<br>Greatest EAH at every age and greatest increase overtime among girls whose both parents had an excess weight	NIH	Not reported	***
Mallan et al.<br>2014	**n**=37<br>**Age range:** 3.5-4 yrs<br>**Mean age:** 3.90 ± 0.04 yrs<br>**Sex:** 57% female<br>**Race/Ethnicity:** Not reported	**Setting:** Home, led by mother<br>**Location:** Australia<br>**Pre-load:** Semi-standardized lunch<br>**Free access** (15 min after)**:** 5 palatable snacks (15 min)<br>**Operationalized EAH measure:** kJ	Adiposity (BMI z-score adjusted for age and sex≥ 85 percentile)<br>Age<br>Age at introduction of solid foods<br>Birthweight z-score<br>Breastfeeding exposure (duration)<br>Eating behavior at 2 yrs (satiety responsiveness, food responsiveness, enjoyment of food, and slowness in eating)<br>Sex	Maternal age at delivery<br>Maternal education	No significant associations with EAH	Australian National Health and Medical Research Council<br>HJ Heinz Meat & Livestock Australia<br>Department of Health South Australia Food Standards<br>Australia New Zealand Queensland University of Technology	Not reported	**

Rollins et al.<br>2014	**n**=180<br>**Age:** 5 yrs<br>**Mean age:** 5.4 ± 0.4 yrs<br>**Sex:** 100% female<br>**Race/Ethnicity:** 100% white<br>Baseline, 7 yrs	**Setting:** Laboratory<br>**Location:** PA, United States<br>**Pre-load:** Self-selected lunch<br>**Free access:** 10 palatable snacks (10 min)<br>**Operationalized EAH measure:** % of total amount of calories available.	Age<br>Temperament at 7yrs (approach and inhibitory control)	Maternal BMI<br>Maternal controlling feeding profile (unlimited Access to Snacks, Set Limits+Does Not Restrict Snacks, Sets Limits+Restricts High Fat/Sugar Snacks or Sets Limits+Restricts All Snacks)	Association between less EAH at 5yrs and higher maternal BMI<br>Greater EAH among girls whose mother restrict all snacks compared to girls whose mother give them unlimited access to snacks, at baseline<br>Association between higher increased in EAH over time and restricting access to all snacks in girls with lower inhibitory control when compared to girls with higher inhibitory control<br>Greater EAH increase over time among girls whose mother had the highest controlling profile compared to those whose mother had a less controlling profile, in girls with lower inhibitory control	NIH	None	***
Farrow et al.<br>2015	**n**=35<br>**Age range:** 5-7yrs<br>**Mean age:** 6.15 ± 0.56 yrs<br>**Sex:** 54% female<br>**Race/Ethnicity:** 91% white	**Setting:** Laboratory<br>**Location:** United Kingdom<br>**Pre-load:** Standardized lunch<br>**Free access:** 4 palatable snacks 2 fruits and vegetables snacks (4 min)<br>**Operationalized EAH measure:** kcal	Adiposity (BMI z-score adjusted for age and sex)<br>Age<br>Experimental group with negative mood induction or control group<br>Sex	Maternal BMI<br>Maternal education<br>Parental child-feeding practices at 3-5 yrs (use of food for emotion regulation, pressure to eat, restriction for weight or	Positive association between EAH for cookies and maternal BMI<br>Positive effect of experimental condition on EAH<br>Associations between greater EAH and lower parental use of food as a rewards and higher parental use of restrictive feeding practices	No external funding	None	***

				health, use of food as a reward)	for health reasons, in the control group<br>Opposite effect on EAH in the experimental group			
Balantekin et al.<br>2017	**n**=158<br>**Age:** 7 yrs<br>**Sex:** 100% female<br>**Race/Ethnicity:** non-Hispanic white	**Setting:** Laboratory<br>**Location:** PA, United States<br>**Pre-load:** Standardized lunch<br>**Free access:** 10 palatable snacks (10 min)<br>**Operationalized EAH measure:** kcal	Adiposity (BMI percentile)	None	Greater EAH among children with a higher BMI percentile (significance not reported)	Not reported	Not reported	**
Fogel et al. 2018	**n**=158<br>**Age:** 4.5 yrs<br>**Sex:** 47% female<br>**Race/Ethnicity:** 59% Chinese, 20% Malay, 20% Indian.<br>Baseline, 6 yrs	**Setting:** Laboratory<br>**Location:** Singapore<br>**Pre-load:** (30 min)<br>Baseline: Buffet lunch<br>Follow-up: Vegetarian fried rice self served<br>**Free access** (25 min later)**:** 4 palatable snacks (5 min)<br>**Operationalized EAH measure:** kcal	Adiposity (BMI z-score adjusted for age and sex ≥ 85 percentile, sum of skinfold thickness measures and waist-to-height ratio)<br>Age<br>Ideal portion size	None	EAH was stable over time, but more children engaged in some EAH at 6 yrs, compared to 4,5 yrs	Singapore National Research Foundation<br>Singapore Institute for Clinical Sciences A*STAR and Nestec SA<br>National Institute for Health Research Southampton Biomedical Research<br>Centre European Union's Seventh Framework Programme, project Early Nutrition	Speaker at conference sponsored by companies selling nutritional products.<br>Part of a consortium with funding from Abbott Nutrition, Nestec and Danone.<br>Employee of Nestec SA (Nestlé Research Center)	***

Galindo et al. 2018	**n**=138<br>**Age range:** 4-5 yrs<br>**Mean age:** 4.7 ± 0,5 yrs<br>**Sex:** 46% female<br>**Ethnicity:** 100% Hispanic<br>Baseline, 18-month follow-up	**Setting:** Laboratory<br>**Location:** TX, United States<br>**Pre-load:** Standardized meal<br>**Free access:** 7 palatable snacks (10 min)<br>**Operationalized EAH measure:** kcal	Adiposity (BMI z-score adjusted for age and sex ≥ 85 percentile)<br>Age	Maternal pressure to eat (self-reported pressure to eat, observed pressure to eat, and observed prompt to eat a different food)	Negative correlation between pressure to eat at baseline and EAH at baseline and 18 months later<br>Positive association between EAH and age<br>Adiposity was correlated with EAH	NICHD<br>US Department of Agriculture/Agricultural Research Service	Not reported	***
Miller et al. 2018	**n**=207, from low-income families<br>**Mean age:** 4.3 ± 0.5 yrs<br>**Sex:** 47% female<br>**Race/Ethnicity:** 55% non-Hispanic white, 19% biracial, 15% black, 10% Hispanic, 1% Asian, 0,5 % American Indian/Alaskan Native<br>Baseline and ~3yrs follow-up (Mean age: 7.9 ± 0.7 yrs)	**Setting:** Preschool<br>**Location:** MI, United States<br>Baseline<br>**Pre-load:** Non-standardized breakfast<br>**Free access:** 6 palatable snacks (10 min)<br>Follow-up<br>**Pre-load:** standardized dinner<br>**Free access:** 6 sweet deserts (5 min)<br>**Operationalized EAH measure:** % of daily caloric need	Adiposity (BMI z-score adjusted for age and sex)<br>Age<br>Child stress exposure index score (based on maternal report of proximal parenting, family level and social-contextual stress indices)	None	EAH increased between follow-up and baseline<br>Cross-sectional positive associations between EAH at both time point and adiposity<br>Positive association between EAH increase over time and overall baseline stress exposure	American Heart Association<br>NIH	None	**
Pang et al. 2020	**n**= 363[2]<br>**Age:** 4.5yrs<br>**Sex:** 49% female<br>**Race/Ethnicity:** Not reported	**Setting:** Laboratory<br>**Location:** Singapore<br>**Pre-load:** Buffet lunch (30min)<br>**Pre-load:** (30 min)	Breastfeeding exposure (low, intermediate, or high, based on duration)	None	No signification association with EAH, at both time points	Singapore National Research Foundation<br>UK Medical Research Council	Speakers at conferences sponsored by companies selling	***

	Baseline and 6yrs (n=311)	Baseline: Buffet lunch<br>Follow-up: Vegetarian fried rice self served<br>**Free access** (15 min later)**:** 4 palatable snacks (5 min)<br>**Operationalized EAH measure:** kcal				National Institute for Health Research<br>National Institute for Health Research Southampton Biomedical Research Centre<br>European Union's Erasmus + Capacity-Building ENeA SEA Project and Seventh Framework Programme project Early Nutrition and ODIN<br>Singapore Institute for Clinical Sciences<br>Agency for Science Technology and Research (A*STAR)	nutritional products.<br>Part of a consortium with funding from Abbott Nutrition, Nestec and Danone.<br>Serves on the scientific advisory council for KerryTaste and Nutrition	
Giuliani & Kelly<br>2021	**n**=47<br>**Age range:** 3.78-6.83 yrs<br>**Mean age:** 4.93 ± 0.86 yrs<br>**Sex:** 49% female<br>**Race/Ethnicity:** 87% Caucasian,	**Setting:** Laboratory<br>**Location:** OR, United States<br>**Pre-load:** Buffet lunch<br>**Free access** (immediately after)**:** 5 palatable snacks (15 min)	Temperament<br>(appetitive self-regulation (delay of gratification task), attentional control, and inhibitory control)	None	Positive association between appetite self-regulation and EAH 1 yr later<br>Highest EAH among children with both lower inhibitory control and lower appetitive self-regulation	Medical Research Foundation of Oregon	None	**

	2% Asian, 9% mixed, 2 % Native American/Indian	**Operationalized EAH measure:** kcal						
**Intervention studies**								
Fisher et al. 2003	**n**=24[2] **Age range:** 2.9-5.1 yrs **Mean age:** 4.0 ± 0.5 yrs **Sex:** 51% female **Race/Ethnicity:** 80% non-Hispanic white, 11% Asian, 6% Hispanic, 3% African American	**Setting:** Laboratory **Location:** PA, United States **Pre-load:** Standardized lunch (15 min) **Free access:** 10 palatable snacks (10 min) **Operationalized EAH measure:** kcal	Children's responses in repeated exposure (4 weeks) to greater portion size of an entrée (change in entrée intake, total energy intake at lunch, bite frequency, bite size and self-served portion size)	None	Positive association between EAH and children's increase in entrée intake	U.S Department of Agriculture	Not reported	**
Boutelle et al. 2011	**n**=36 children with excess weight or obesity and high EAH **Age range:** 8-12 yrs **Mean age:** 10.3 ± 1.3 yrs **Sex:** 58% female **Race/Ethnicity:** 40% Caucasian, 17% African American, 23% mixed, 20% other Baseline, post-treatment, and 6 months follow-up	**Setting:** Laboratory **Location:** United States **Pre-load:** Standardized pizza dinner **Free access** (10 min later)**:** 10 palatable snacks (10 min) **Operationalized EAH measure:** % of caloric needs	Treatment condition for 8 weeks food cue exposure treatment or appetite awareness training	None	Decreased EAH after the food cue exposure treatment and 6 months post-treatment Significant differences in EAH between the two conditions at post-treatment No effect of appetite awareness training on children's EAH	University of Minnesota Faculty Development	Not reported	***

Boutelle et al.<br>2014 (May)	**n**=29 children with excess weight or obesity and high EAH<br>**Age range:** 8-12 yrs<br>**Mean age:** 11.0 ± 1.2 yrs<br>**Sex:** 46% female<br>**Race/Ethnicity:** 54% non-Hispanic white<br>Baseline, posttreatment	**Setting:** Laboratory<br>**Location:** CA, United States<br>**Pre-load:** Home meal (presumed). Children were given a snack if not full.<br>**Free access**: 11 palatable snacks (10 min)<br>**Operationalized EAH measure:** % of caloric needs and kcal	Treatment condition for one session<br>Attention Modification Program (AMP)<br>or<br>Attention Control condition (ACC)	None	Increased EAH after training session in the ACC group when compared to AMP group<br>Similar EAH over time in the AMP group	NIH	Not reported	**
Lee et al.<br>2017	**n**=29<br>**Age range:** 3-5 yrs<br>**Mean age:** Not reported<br>**Sex:** Not reported<br>**Race/Ethnicity:** Not reported	**Setting:** Early care and educational centre<br>**Location:** TX, United States<br>**Pre-load:** Meal provided by the center<br>**Free access**: 2 snacks palatable snacks (10 min)<br>**Operationalized EAH measure:** kcal	12-hour intervention on healthy eating and physical activity	None	Increased EAH after the intervention when compared to baseline<br>After adjusting for center, the increase in EAH didn't remain significant	NICHD	None	*
	**n**=41<br>**Age range:** 3-5 yrs<br>**Mean age:** 3.9 ± 0.72 yrs<br>**Sex:** 45% female<br>**Race/Ethnicity:** 65% Hispanic, 10% Asian, 10%	**Setting:** Early care and educational centre<br>**Location:** AZ, United States<br>**Pre-load:** Meal provided by the center<br>**Free access**: 2 snacks palatable snacks (10 min)						

	African American, 5% white, 5% Native American, 5% other	**Operationalized EAH measure:** kcal						
Rhee et al. 2019	**n**=91<br>**Age range:** 4-6 yrs<br>**Mean age:** 5.11 ± 0.73 yrs<br>**Sex:** 46% female<br>**Race/Ethnicity:** 55% Caucasian, 17% Asian/Pacific Islander, 14% African/American, 14% mixed/other, 45% Hispanic	**Setting:** Preschool<br>**Location:** CA, United States<br>**Pre-load:** Not reported. If children were still hungry, they were offered a granola bar<br>**Free access**: 10 palatable snacks (10 min)<br>**Operationalized EAH measure:** kcal<br>Baseline and post-intervention	Intervention group (activities centered on healthy eating strategies including inhibitory training towards energy-dense snack foods) or Control group (activities centered around dental hygiene, good sleep habit/routine, and physical activities)<br>Adiposity (BMI z-score adjusted for age and sex ≥85 percentile)	None	Increased EAH for both group from baseline to post intervention with no between-group differences<br><br>Positive association between EAH and adiposity in the control group, at post intervention	NICHD	None	****
Savage et al. 2020	**n**=35<br>**Age range:** 6-9 yrs<br>**Mean age:** 7.7 ± 1.1 yrs<br>**Sex:** Not reported<br>**Race/Ethnicity:** Predominantly	**Setting:** Laboratory<br>**Location:** PA, United States<br>**Pre-load:** Standardized lunch (20 min)<br>**Free access**: 11 palatable snacks (7 min)	Eight experimental conditions using a multiphase optimization strategy for 4 weeks (parent shared decision making, child mindfulness,	None	No differences in EAH at follow-up between experimental conditions	U.S Department of Agriculture National Institute for Food and Agriculture<br>National Confectioners Association	Personal fees from Let us Go Maine, American Academy of Paediatrics, Danone Happy Baby Organics	**

	non-Hispanic white	**Operationalized EAH measure:** kcal<br>Post-intervention only	child attention control, and home supply of candies)				and grants from NIH<br>Grants, personal fees and non-financial support from National Confectioner's Association.	
Morrison et al.<br>2021	**n**=93<br>**Age range:** 8-12 yrs<br>**Mean age:** 10.0 ± 1.4 yrs<br>**Sex:** 54% female<br>**Race/Ethnicity:** 77% European/other, 15% Maori, 3% pacific and 4% Asian	**Setting:** Laboratory (presumed)<br>**Location:** New Zealand<br>**Pre-load:** Buffet dinner<br>**Free access** (15 min later)**:** 12 palatable snacks (15 min)<br>**Operationalized EAH measure:** kcal	Age<br>Experimental effect (cross-over design): sleep restriction and sleep extension<br>Sex<br>Adisposity (BMI z-score adjusted for age and sex ≥ 85 percentile)	None	Less EAH after the second experiment, regardless of experimental group (learning effect)	Royal Society of New Zealand<br>University of Chicago Grant<br>Karitane Fellowship	None	****

kcal: kilocalories; kJ: kilojoules; EAH: eating in the absence of hunger; BMI: Body mass index; NIH: National Institutes of Health Research; NICHD: Eunice Kennedy Shriver National Institute of Child Health and Human Development; FONDECYT: National Fund of Scientific and Technological Development; NIDDK: National Institute of Diabetes and Digestive and Kidney Disease.

[1]Quality assessment's legend: * weak; ** moderate; *** strong; **** very strong.

[2]The number of participants represent the ones who completed the EAH assessment, but only characteristics for the whole study sample were available.

[3]We considered the delay of gratification task as a measure of appetitive self-regulation even if the authors considered it a measure of non-food related self-regulation.

Table S2. Characteristics of included studies assessing eating in the absence of hunger (EAH) during adolescence (>12 years old) with the laboratory protocol

Authors, publication date	Sample size and characteristics	EAH assessment method	Child measures	Familial measures	Associations with EAH	Funding sources	Conflicts of interest	Quality assessment[1]
**Cross-sectional studies**								
Shomaker et al. 2010 (August)	**n**=78<br>**Age range:** 13-17 yrs<br>**Mean age:** 15.2 ± 1.4 yrs<br>**Sex:** 44% female<br>**Race/Ethnicity:** 47% non-Hispanic white	**Setting:** Laboratory<br>**Location:** MD, United States<br>**Pre-load:** 2 separates days; standardized lunch and buffet meal<br>**Free access:** 12 palatable snacks (15 min)<br>**Operationalized EAH measure:** kcal	Adiposity (BMI z-score adjusted for age and sex ≥85 percentile, overweight or obese and normal weight, fat free mass, % Fat mass)<br>Age<br>Pubertal status<br>Race<br>Sex	None	Positive association between EAH and pubertal status<br>Positive association between EAH and weight status as well as % fat mass	National Research Service Award and Intramural Research Program from NICHD<br>NIDDK Uniformed Services, University of the Health Sciences<br>NIMHD, the OBSSR	None	***
Reina et al. 2013	**n**=90<br>**Age range:** 13-17 yrs<br>**Mean age:** 15.27 ± 1.39 yrs<br>**Sex:** 48% female<br>**Race/Ethnicity:** 47% non-Hispanic white, 40% non-Hispanic black, 7% Asian, 3% Hispanic, 3% mixed	**Setting:** Laboratory<br>**Location:** MD, United States<br>**Pre-load:** 2 separates days; standardized lunch and buffet meal<br>**Free access:** 12 palatable snacks (15 min)<br>**Operationalized EAH measure:** kcal	Body image (appearance orientation, overweight preoccupation and appearance evaluation)<br>Sex<br>Pressure from media (SATAQ-3)	Parental child-feeding practices (restriction, concern about eating and monitoring)	Positive association between EAH and parental restriction towards food, and family pressure to be thin<br>Positive association between EAH and higher media pressure to be thin and higher uses of media as a source of information	National Research Service Award<br>Intramural Research Program grant and Pathway to Independence Award from NICHD<br>NIMHD, OBSSR<br>NIH Clinical Center Bench to Bedside Award	Not reported	****

			Pressure to be thin (from family, friends and media)<br>Uses of media for information on appearance		for appearance, for girls only<br>Positive indirect associations between EAH and parental restriction towards foods, parental concern about eating, sociocultural pressure to be thin and use of media for information, via appearance orientation and overweight preoccupation<br>Positive indirect association between EAH and parental monitoring, via overweight preoccupation<br>Positive indirect associations between EAH and use of media for information and pressure to be thin from media via appearance orientation and overweight preoccupation, for girls only			
Kelly et al.<br>2015 (August)	**n**=182<br>**Age range:** 13-17 yrs<br>**Mean age:** 15.3 ± 1.4 yrs<br>**Sex:** 65% female	**Setting:** Laboratory<br>**Location:** MD, United states<br>**Pre-load:** Buffet lunch<br>**Free access:** 12 palatable snacks (15 min)	Adiposity (fat-free mass)<br>Depressive symptoms<br>Dietary restraint	None	Positive association between EAH and dietary restraint, in girls only<br>Greater EAH among boys compared to girls	NIH Intramural Research Program from NICHD<br>NIH Bench to Bedside Program, OBSSR	Not reported	***

	**Race/Ethnicity:** 56% non-Hispanic white	**Operationalized EAH measure:** kcal	Experimental group with sad mood induction (sad film) or control group (neutral film)<br>Sex		Positive association between EAH and fat-free mass	NIH National Research Service Award<br>Pathway to Independence Award, NICHD		
Kelly et al. 2016	**n**=119 at risk for T2D<br>**Age range:** 12-17 yrs<br>**Mean age:** 14.5 ± 1.6 yrs<br>**Sex:** 100% female<br>**Ethnicity:** 62% non-Hispanic black/African American, 16% non-Hispanic white, 11% Hispanic, 8% mixed and 3% Asian	**Setting:** Laboratory<br>**Location:** MD, United States<br>**Pre-load:** Buffet meal<br>**Free access:** Number of snacks not reported (15 min)<br>**Operationalized EAH measure:** kcal	Daytime sleepiness<br>Sleep duration	None	No significant association with EAH	NICHD<br>NIH Intramural Research Program from NICHD<br>NIH Bench to Bedside Program, the Office of Disease Prevention<br>NIH, the OBSSR	None	***
Radin et al. 2016	**n**=178<br>**Age range:** 13-17 yrs<br>**Mean age:** 15.3 ± 1.4 yrs<br>**Sex:** 66% female<br>**Race/Ethnicity:** 57% non-Hispanic white, 26% non-Hispanic black, 7% Hispanic,	**Setting:** Laboratory<br>**Location:** MD, United States<br>**Pre-load:** Buffet lunch<br>**Free access:** 12 palatable snacks (15 min)<br>**Operationalized EAH measure:** kcal	Experimental group with sad mood induction (sad film) or control group (neutral film)<br>LOC (past month and current)	None	Positive association between EAH and LOC eating during test meal	NIH National Research Service Award Pathway to Independence Award and NIH Intramural Research Program grant from NICHD<br>NIH Bench to Bedside Program, OBSSR	None	***

	6% mixed or other and 6% Asian							
Annameier et al. 2018	**n=** 107 at risk of T2D.<br>**Age range:** 12-17 yrs<br>**Mean age:** 14.53 ± 1.63 yrs<br>**Sex:** 100% females<br>**Race/Ethnicity:** 61,6 % Non-Hispanic Black	**Setting:** Laboratory<br>**Location:** DC, United States<br>**Pre-load:** Buffet lunch<br>**Free access** (60 min after initiation of lunch meal)**:** number of snacks not reported (15 min)<br>**Operationalized EAH measure:** kcal	Eating behavior<br>(Disposable mindfulness and LOC)	None	Negative association between EAH and mindfulness	NICHD<br>NIH Bench to Bedside Program OBSSR<br>NIH Office of Disease Prevention	None	***
Blanco et al. 2019	**n=** 605<br>**Mean age:** 16.7 yrs<br>**Sex:** 48% females.<br>**Race/Ethnicity:** 100% Chilean	**Setting:** Laboratory<br>**Location:** Chile<br>**Pre-load:** Standardized breakfast<br>**Free access** (20 min later)**:** 8 palatable snacks (20 min)<br>**Operationalized EAH measure:** kcal	Adiposity (BMI z-score adjusted for age and sex ≥ 85 percentile)<br>Sex	None	Negative association between EAH and adiposity	NHLBI<br>NICHD	None	****

Obregon et al. 2020	**n=** 38<br>**Age range:** 8-14 yrs<br>**Mean age:** 12.6 ± 1.45 yrs<br>**Sex:** not reported<br>**Race/Ethnicity:** not reported	**Setting:** Laboratory<br>**Location:** Chile<br>**Pre-load:** Standardized lunch (20 min)<br>**Free access:** 9 palatable snacks with mayonnaise and ketchup<br>**Operationalized EAH measure:** g and kcal	Dopamine D2 receptor rs1800497 genotype (TaqI A1 allele carriers and non-carriers)	None	No significant association with EAH	National Scientific and Technological Development fund<br>Comision Nacional de Investigacion Cientifica Y Tecnologia	None	**
**Prospective cohort studies**								
Reyes et al. 2014	**n=** 576<br>**Age range:** 16-17 yrs<br>**Mean age:** 16.7 ± 0.2 yrs<br>**Sex:** 48% female<br>**Race/Ethnicity:** 100% Chilean	**Setting:** Laboratory<br>**Location:** Chile<br>**Pre-load:** Standardized breakfast<br>**Free access:** 7 palatable snacks (20 min)<br>**Operationalized EAH measure:** kcal	Adiposity (BMI z-score adjusted for age and sex ≥85 percentile)<br>Sex	Child-Breastfeeding duration (< or > 6 months)	Lower EAH among adolescents who were breastfed past 6 months	NHLBI<br>NICHD<br>National Center of Minority Health and Health Disparities	None	***
**Intervention studies**								
Schyns et al. 2018	**n=**40 with overweight<br>**Age range:** 12-18 yrs<br>**Mean age:** 14.38 ± 1.84 yrs<br>**Sex:** 60% female<br>**Ethnicity:** not reported	**Setting:** Laboratory<br>**Location:** Netherlands<br>**Pre-load:** Sandwich meal<br>**Free access** (60 min after initiation of lunch meal)**:** 6 sweet deserts (10 min)<br>**Operationalized EAH measure:** % of daily caloric need and kcal	Experimental group (food cue exposure, with one food item from the EAH assessment) vs Control group (lifestyle intervention)<br>Habituation of food cues reactivity (desire to eat	None	Less EAH of the exposed food and the non-exposed food in the experimental group, compared to the control group<br>Within session habituation of desire to eat was related to more EAH of the non-exposed food item	Netherlands Organisation for Scientific Research	None	**

		and salivation) within session and between session	

kcal: kilocalories; EAH: eating in the absence of hunger; T2D: type 2 diabetes; LOC: Loss of control eating; BMI: Body Mass Index; NHLBI: National Heart, Lung, and Blood Institute; OBSSR: Office of Behavioral and Social Sciences Research; NIH: National Institutes of Health; NICHD: Eunice Kennedy Shriver National Institute of Child Health and Human Development; NIDDK: National Institute of Diabetes and Digestive and Kidney Disease; NIMHD: National Institute on Minority Health and Health Disparities.

[1] Quality assessment's legend: * weak; ** moderate; *** strong; **** very strong.

Table S3. Characteristics of included studies assessing EAH during childhood (≤12 years old) with the questionnaire

Authors, publication date	Sample size and characteristics	EAH assessment method	Child measures	Familial measures	Associations with EAH	Funding sources	Conflicts of interest	Quality assessment[1]
**Cross-sectional studies**								
Gill et al. 2014	**n=** 145<br>**Age range:** 35 months-25 yrs<br>**Mean age:** 9.7 ± 4.2 yrs<br>**Sex:** 48% female<br>**Race/Ethnicity:** Not reported	**Setting:** Laboratory<br>**Location:** Various locations in the United States<br>**Questionnaire:** EAH-P, all subscales	Adiposity (BMI z-score adjusted for age and sex)<br>Age<br>16p11.2 genotype (deletion carriers, duplication carriers and noncarriers; *de novo* or not)<br>Full-scale intelligence quotient<br>Sex	Parental child-feeding practices (restriction and pressure to eat)	Positive association between all subscale of EAH-P and adiposity, in *de novo* deletion families<br>Positive association between all subscale of EAH-P and adiposity, in the whole deletion carriers sample<br>Negative association between EAH due external cues and boredom and pressure to eat<br>Positive association between parental use of restriction and EAH due to boredom, fatigue, and external cues<br>Positive association of 16p11.2 deletion genotype and EAH due to external cues and due to boredom	Simons Variation in Individual Project<br>Canadian Institutes of Health Research	None	***
Kral et al. 2017	**n=** 50<br>**Age range:** 8-10 yrs<br>**Mean age:** 9.6 ± 0.8 yrs<br>**Sex:** 52% female<br>**Race/Ethnicity:** 68% African American, 22% Caucasian, 10%	**Setting:** Laboratory<br>**Location:** PA, United States<br>**Questionnaire:** EAH-P, all subscales	None	Household food security	Negative association between EAH due to external cues and household food security	NIH	None	**

	mixed (8% Hispanic / 77% non-Hispanic)							
Obregon et al. 2017 (February)	**n=** 258<br>**Age range:** 8-14 yrs<br>**Mean age:** 11.4 ± 1.6 yrs<br>**Sex:** 44% female<br>**Race/Ethnicity:** Not reported	**Setting:** Laboratory<br>**Location:** Chile<br>**Questionnaire:** EAH-C, all subscales	Adiposity (BMI z-score adjusted for age and sex ≥ 85 percentile)<br>MC4R rs17782313 genotype (C-allele carriers and non-carriers)	None	No significant association with EAH	FONDECYT<br>Comision Nacional de Investigacion Cientifica Y Tecnologia	None	***
Obregon et al. 2017 (March)	**n=** 258<br>**Age range:** 8-14 yrs<br>**Mean age:** 11.4 ± 1.6 yrs<br>**Sex:** 44% female<br>**Race/Ethnicity:** Not reported	**Setting:** Laboratory<br>**Location:** Chile<br>**Questionnaire:** EAH-C, all subscales	Adiposity (BMI z-score adjusted for age and sex ≥ 85 percentile)<br>Dopamine D2 receptor rs1800497 genotype (TaqI A1 allele carriers and non-carriers)<br>Sex	None	Positive association between EAH due to fatigue/boredom or external cues and adiposity	FONDECYT<br>Comision Nacional de Investigacion Cientifica Y Tecnologia	None	***
Obregon et al. 2018	**n=** 258<br>**Age range:** 8-14 yrs<br>**Mean age:** 11.4 ± 1.6 yrs<br>**Sex:** 44% female<br>**Race/Ethnicity:** Not reported	**Setting:** Laboratory<br>**Location:** Chile<br>**Questionnaire:** EAH-P, all subscales	Adiposity (BMI z-score adjusted for age and sex ≥ 85 percentile)<br>Rs9939609 genotype (A allele carriers / non-carriers)<br>Sex	None	Greater EAH among boys who carried the A allele, in children with normal weight	FONDECYT<br>Comision Nacional de Investigacion Cientifica Y Tecnologia	None	***

EAH: eating in the absence of hunger; EAH-P: eating in the absence of hunger questionnaire for children and adolescents, parent-report; NIH: National Institutes of Health Research; FONDECYT: National Fund of Scientific and Technological Development.

[1]Quality assessment's legend: * weak; ** moderate; *** strong; **** very strong.

Table S4. Characteristics of included studies assessing eating in the absence of hunger (EAH) during adolescence (>12 years old) with the questionnaire

Authors, publication date	Sample size and characteristics	EAH assessment method	Child measures	Familial measures	Associations with EAH	Funding sources	Conflicts of interest	Quality assessment[1]
**Cross-sectional studies**								
Kral et al. 2010	**n**=31<br>**Age:** 13 yrs<br>**Sex:** 48% female<br>**Ethnicity:** 100% Caucasian	**Setting:** Laboratory<br>**Location:** PA, United States<br>**Questionnaire:** EAH-C, all subscales	Adiposity (BMI z-score adjusted for age and sex ≥ 85 percentile)<br>Sex	Familial risk for obesity (based on mother's pre-pregnancy BMI)	Greater EAH among girls at low risk for obesity compared to girls at high risk and boys at low risk or high risk for obesity<br>Negative association between EAH and adiposity, for girls only<br>Greater EAH for girls compared to boys, in normal weight children only	National Center for Research resources	None	**
Shomaker et al. 2010 (November)	**n**=367<br>**Age range:** 6-17 yrs<br>**Mean age:** 12.7 ± 2.8 yrs.<br>**Sex:** 48% female<br>**Race/Ethnicity:** 58% non-Hispanic white	**Setting:** Laboratory<br>**Location:** MD, United States<br>**Questionnaire:** EAH-C all subscales and EAH-P all subscales	Binge eating and Loss of control (LOC) eating (Objective binge eating and subjective binge-eating, objective overeating without a sense of LOC and no reported episodes of overeating)	None	Greater EAH due to negative affect in participants reporting binge eating compared to participants reporting objective overeating without a sense of LOC or no episode and greater EAH due to external cues and fatigue/boredom compared to those who do not report any episode<br>Greater EAH due to fatigue/boredom among participants reporting subjective binge eating compared to participants reporting objective overeating without a sense of LOC<br>Greater parental report of EAH due to negative affect and	NIH Intramural Research Program and National Research Service Award from NICHD<br>NIMHD<br>OBSSR	None	***

					fatigue/boredom among children reporting any eating episode compared to no reported episode<br>Greatest parental report of EAH due to external cues among children who overeat without a sense of LOC<br>Greater parental report of EAH due to external cues among children who overeat without a sense of LOC compared to children reporting objective binge eating episode and no overeating episode<br>Higher EAH du to external cues among children reporting subjective binge eating compared to children reporting no overeating episode			
Zocca et al. 2011	**n**=305<br>**Age range:** 8-17 yrs<br>**Mean age:** 13.62 ± 2.65 yrs.<br>**Sex:** 50% female<br>**Race/Ethnicity:** 54% non-Hispanic white, 34% African American, 12% other	**Setting:** Laboratory<br>**Location:** MD, United States<br>**Questionnaire:** EAH-C all subscales and EAH-P all subscales	Adiposity (BMI z-score adjusted for age and sex ≥ 85 percentile)<br>Age<br>LOC	Maternal binge eating<br>Maternal BMI<br>Maternal EAH	Positive association between children's EAH and children's LOC, adiposity and maternal binge eating; stronger association for pre-adolescents (<13 yrs) compared to adolescents (≥13 yrs)<br>Positive association between children's EAH and maternal EAH; stronger for adolescents compared to pre-adolescents	NIH Intramural Research Program and National Research Service Award from NICHD<br>NIMHD<br>OBSSR<br>Uniformed Services University of the Health Sciences<br>NIDDK	An author is a commissioned Officer of the United Sates Public Health Service, Department of Health and Human Services	***

Pivarunas et al. 2015	**n**=114 at risk for type 2 diabetes<br>**Age range:** 12-17 yrs<br>**Mean age:** 14.5 ± 1.6 yrs.<br>**Sex:** 100% female<br>**Race/Ethnicity:** 61% non-Hispanic black, 18% non-Hispanic white, 12% Hispanic, 11% other	**Setting:** Laboratory<br>**Location:** MD, United States<br>**Questionnaire:** EAH-C, all subscales	Mindfulness	None	Negative association between EAH due to fatigue/boredom and mindfulness	NICHD<br>NIH Intramural Research Program from NICHD<br>NIH Bench to Bedside Program, the NIH, the Office of Disease Prevention<br>OBSSR, NIH Office of Intramural Training and Education.	Not reported	***
Lemay-Russel et al. 2019	**n**=123<br>**Age range:** 8-17 yrs<br>**Mean age:** 12.7 ± 2.6 yrs.<br>**Sex:** 52% female<br>**Race/Ethnicity:** 44% non-Hispanic white, 29% black or African American, 11% Asian, 7% Hispanic/Latino, 7% mixed/other, 0.8% unknown	**Setting:** Laboratory<br>**Location:** MD, United States<br>**Questionnaire:** EAH-C, total score	Adiposity (Fat mass)<br>Sleep duration (weekly, weeknight and weekend averages and weekend catch-up sleep)<br>Wake time and bedtime (weekday vs weekend)	None	Negative association between EAH and sleep duration on weeknights<br>Positive association between EAH and catch-up sleep duration on weekends	Intramural Research Program from the NICHD	None	***
Shank et al. 2019	**n**=200<br>**Age range:** 8-17 yrs<br>**Mean age:** 13.1 ± 2.8 yrs<br>**Sex:** 54% female	**Setting:** Laboratory<br>**Location:** MD, United States	Alexithymia<br>Sex	None	Higher EAH among girls compared to boys<br>Positive association between EAH and alexithymia	Intramural Research Program from the NICHD<br>NIH OBSSR	An author is a commissioned Officer of the United	****

	**Race/Ethnicity:** 45% non-Hispanic white, 29% non-Hispanic black, 9% Hispanic/Latino, 18% mixed/other/unknown	**Questionnaire:** EAH-C, total score					Sates Public Health Service	
Rubin et al. 2021	**n**=201<br>**Age range:** 8-17 yrs<br>**Mean age:** 13.1 ± 2.8 yrs.<br>**Sex:** 54% female<br>**Race/Ethnicity:** 48% white, 30% black/African American, 11% Asian, 11% mixed/other, 9% Hispanic/Latino	**Setting:** Laboratory<br>**Location:** MD, United States<br>**Questionnaire:** EAH-C, total score	Age<br>Emotional eating<br>Negative Affect<br>Sex<br>Weight-based teasing perception	None	Positive indirect association between EAH and weight-based teasing via negative affect<br>Positive association between EAH and age<br>Greater EAH among girls than boys<br>Positive association between EAH and emotional eating	Intramural Research Program from the NICHD	None	***
**Prospective cohort studies**								
Shapiro et al. 2017	**n**=268<br>**Age range:** 12-19 yrs<br>**Mean age:** 16.1 ± 1.0 yrs<br>**Sex:** 49% female<br>**Race/Ethnicity:** 41% non-Hispanic white, 10% non-Hispanic black, 42% Hispanic, 6% other	**Setting:** Laboratory<br>**Location:** CO, United States<br>**Questionnaire:** EAH-C, all subscales and total score	Daily energy intake<br>Sex<br>Exposure to maternal gestational diabetes *in utero*	None	Greater EAH due to negative affect among girls compared to boys<br>Positive association between EAH (all subscales and total score) and daily energy intake<br>Positive association between EAH-C total score and *in utero* exposure to gestational diabetes, for girls compared to all other participants and to exposed boys<br>Greater EAH due to fatigue/boredom for girls exposed to maternal gestational diabetes compared to all other participants	NIDDK	Not reported	****
Derks et al. 2019	**n**=1097<br>**Age:** 13 yrs	**Setting:** Laboratory	Adiposity (BMI z-score adjusted for age and sex ≥	Maternal gestational glucose	Higher EAH for girls exposed *in utero* to maternal isolated hyperglycemia or impaired	NIH	None	**

	**Mean age:** 13.1 ± 2.8 yrs.<br>**Sex:** 50% female<br>**Race/Ethnicity:** 66% white, 15% black/African American, 11% other, 4% Hispanic, 3% Asian	**Location:** MA, United States<br>**Questionnaire:** Two modified questions from the EAH-C	85 percentile, fat mass index, fat free mass and %body fat)<br>Adiposity in mid childhood: (BMI z-score adjusted for age and sex ≥ 85 percentile)<br>Consumption of energy-dense, low nutritive foods<br>Sex<br>Sugar sweetened beverage intake	tolerance (gestational diabetes mellitus, impaired glucose tolerance, isolated hyperglycem ia, or normal glucose tolerance)<br>Maternal glucose levels after the glucose challenge test	glucose tolerance compared to those exposed to normal glucose tolerance<br>Less EAH for boys exposed *in utero* to maternal impaired glucose tolerance compared to those exposed to normal glucose tolerance<br>Positive associations between EAH in adolescence and adiposity in adolescence (BMI z-score, fat mass index and fat-free mass index), but not with BMI z-score in mid-childhood nor BMI z-score change from childhood to adolescence<br>Positive association between EAH and sugar sweetened beverage intake	Dutch KNAW Ter Meulen Foundation Dutch Diabetes Foundation

EAH: eating in the absence of Hunger; EAH-C: eating in the absence of hunger questionnaire for children and adolescents; EAH-P: eating in the absence of hunger questionnaire for children and adolescents, parent-report; LOC: loss of control eating; BMI: body mass index, NIH: National Institutes of Health, NICHD: Eunice Kennedy Shriver National Institute of Child Health and Human Development; NIDDK: National Institute of Diabetes and Digestive and Kidney Disease; NIMHD: National Institute on Minority Health and Health Disparities; OBSSR: Office of Behavioral and Social Sciences Research.

[1] Quality assessment's legend: * weak; ** moderate; *** strong; **** very strong.

Table S5. Characteristics of included studies assessing eating in the absence of hunger (EAH) during childhood (≤ 12 years old) or adolescence (>12 years old) with the laboratory protocol and the questionnaire

Authors, publication date	Sample size and characteristics	EAH assessment method	Child measures	Familial measures	Associations with EAH	Funding sources	Conflicts of interest	Quality assessment[1]
**Prospective cohort studies**								
Kelly et al. 2015 (July)	**n=** 196<br>**Age range:** 13-17 yrs<br>**Mean age:** 15.3 ± 1.4 yrs<br>**Sex:** 65% female<br>**Ethnicity:** not reported<br>Baseline and 1 yr follow up (n=163)	**Setting:** Laboratory<br>**Location:** DC, United States<br>**Pre-load:** Buffet lunch (20 min)<br>**Free access (30 min after):** 9 palatable snacks (15 min)<br>**Operationalized EAH measure:** kcal<br>**Questionnaire**: EAH-P, external eating subscale	Adiposity (BMI z-score adjusted for age and sex ≥ 85 percentile, BMI, BMI z-score, and fat mass)<br>Age<br>Sex	None	Greater EAH (laboratory) among boys compared to girls, at baseline<br>Lowest EAH (laboratory) among girls with a normal weight at baseline<br>Greater EAH (EAH-P) among participant with an excess weight compared to participant with a normal weight, at baseline<br>Girls with excess weight had a higher EAH-P score when compared boys or girls with normal weight, at baseline<br>Greater EAH (laboratory or EAH-P) among participant with an excess weight compared to participants with a normal weight, at follow-up<br>Greater EAH (EAH-P) among boys and girls with excess weight compared to boys and girls with normal weight, at follow-up<br>Positive association between EAH (laboratory or EAH-P) and BMI and BMI z-score, at baseline	NIH Intramural Research Program from NICHD<br>NIH Bench to Bedside Program<br>OBSSR<br>NIH National Research Service Award<br>Pathway to Independence Award from NICHD	None	***

					Positive association between EAH (EAH-P) and fat mass, at baseline<br>Positive associations between EAH (EAH-P) and BMI, BMI z-score and fat mass, at follow-up.<br>Positive association between EAH (laboratory) and BMI z-score at follow-up<br>EAH (laboratory or EAH-P) was similar between baseline and at the 1-yr follow-up			
**Intervention studies**								
Boutelle et al.<br>2014 (January)	**n=** 44 children with excess weight or obesity and high EAH<br>**Age range:** 8-12 yrs<br>**Mean age:** 10,2 ± 1,3 yrs<br>**Sex:** 50% female<br>**Race/Ethnicity:** 69,1% non-Hispanic white<br>Baseline, post treatment (n=42), and 4-month follow-up (n=39)	**Setting:** Laboratory<br>**Location:** MI, United States<br>**Pre-load:** Standardized pizza dinner<br>**Free access:** (10 min later)**:** 11 palatable snacks (10 min)<br>**Operationalized EAH measure:** % of caloric needs<br>**Questionnaire:** EAH-P, all subscale	Treatment condition (4 months):<br>children appetite awareness and food cue exposure treatment or control (no treatment)	None	Laboratory measure: At 4-month post-treatment, less EAH in the treatment group, when compared to the control group<br>EAH-P: In the treatment condition, EAH-P scores from all subscales decreased post treatment and at 4-month post treatment when compared to baseline	University of Minnesota Faculty Development<br>NIH	None	***

EAH: eating in the absence of hunger; BMI: body mass index; NIH: National Institutes of Health, NICHD: Eunice Kennedy Shriver National Institute of Child Health and Human Development; OBSSR: Office of Behavioral and Social Sciences Research.

[1] Quality assessment's legend: * weak; ** moderate; *** strong; **** very strong.
